# From Modality Performance to Graceful Degradation: A PRISMA 2020 Systematic Review of Sensor Architectures for Autonomous Vehicles

**DOI:** 10.3390/s26165316

**Published:** 2026-08-21

**Authors:** Patrik Viktor

**Affiliations:** Keleti Károly Faculty of Business and Management, Óbuda University, Tavaszmező u. 15-17, 1084 Budapest, Hungary; viktor.patrik@uni-obuda.hu

**Keywords:** autonomous vehicles, sensors, LiDAR, radar, camera, sensor fusion, PRISMA, adverse weather, fault diagnosis, graceful degradation, SOTIF

## Abstract

Autonomous vehicles depend on heterogeneous sensing systems whose performance varies with range, illumination, weather, object material, traffic geometry, contamination, calibration quality, and cyber-physical interference. This PRISMA 2020 and PRISMA-S systematic review synthesized 65 peer-reviewed primary studies selected from 2143 records identified through four databases. After removal of 793 records before screening, 1350 titles and abstracts were screened; 273 full texts were assessed and 208 were excluded with documented reasons. The final evidence base covers cameras, LiDAR, radar, thermal and event cameras, GNSS/IMU localization, calibration, synchronization, multimodal fusion, adverse-weather perception, sensor-health monitoring, and fault-tolerant perception. No modality was universally superior: comparative performance depended on hardware generation, dataset, environmental severity, range, and metric. Direct evidence was strongest for component-level perception and controlled degradation, whereas health-conditioned fusion, ODD restriction, and minimum-risk behavior were supported mainly by partial experimental evidence and safety-oriented synthesis. The review therefore proposes, rather than claims to validate, a reliability-aware architecture that separates sensor health from task confidence, preserves uncertainty and provenance, adapts fusion, and constrains operation when residual evidence is insufficient. The review was retrospectively registered in PROSPERO on 30 July 2026 (CRD420261465869).

## 1. Introduction

Automated driving replaces the continuously adapting perceptual, cognitive, and anticipatory capabilities of a human driver with an engineered chain of sensors, transducers, timing systems, communication interfaces, calibration models, learned representations, probabilistic estimators, digital maps, and decision-making modules. In the SAE taxonomy, increasing levels of driving automation progressively transfer responsibility for the dynamic driving task and, at higher levels, the fallback function from the human driver to the automated-driving system within a predefined operational design domain (ODD) [1]. This transfer of responsibility has fundamental consequences for sensing-system design. A human driver can compensate for incomplete visual information by changing gaze direction, reducing speed, increasing following distance, recalling previous observations, or interpreting subtle contextual cues. An automated-driving system must reproduce these adaptive behaviors through explicit sensing, estimation, uncertainty management, and operational constraints. Consequently, the sensing subsystem cannot be regarded as a peripheral collection of devices. It constitutes the primary evidentiary interface between the physical environment and the automated-driving stack.

Through its sensors, the vehicle must infer free space, lane topology, road boundaries, traffic-control devices, object class, object position, relative and absolute velocity, pedestrian and cyclist motion, surface conditions, ego-motion, and global or local position. These estimates are subsequently transformed into predictions, risk assessments, trajectories, and control actions. Errors introduced at the sensing stage may therefore propagate through every downstream function. A missed pedestrian detection may affect path prediction and emergency braking, while a biased localization estimate may simultaneously distort map alignment, lane association, and trajectory planning. General surveys of automated-driving technology consistently identify environmental perception as one of the principal technical bottlenecks for higher levels of automation [2,3]. Localization and mapping are similarly recognized as safety-critical because perception results can only be interpreted correctly when the position and orientation of the ego vehicle are known with sufficient accuracy [4,5]. More recent reviews further emphasize that the challenge is not limited to nominal detection accuracy but includes uncertainty, computational latency, environmental variability, and the ability to maintain performance outside the distribution represented in training data [6,7].

The central question is therefore not merely whether an individual sensor can detect an object under controlled or favorable conditions. Safety depends on whether the complete sensing architecture can produce sufficiently accurate, timely, and interpretable evidence throughout the intended ODD. This includes ordinary traffic situations as well as rare combinations of illumination, precipitation, road-surface reflectivity, atmospheric obscurants, sensor contamination, dense traffic, construction zones, unusual object geometries, and partial hardware or communication failure. Such combinations are particularly important because sensing degradation is rarely caused by a single isolated variable. Low sunlight may coincide with a wet road surface, spray from a leading vehicle, faded lane markings, and temporary construction signs. Snowfall may reduce camera contrast, modify LiDAR backscatter, obscure road boundaries, and simultaneously degrade GNSS reception through environmental or infrastructural conditions. A safety-relevant evaluation must therefore consider both modality-specific limitations and compound scenarios in which several sources of uncertainty interact.

The established automotive sensor stack combines multiple physical measurement principles. Visible-light cameras provide dense angular resolution, color, texture, object appearance, and semantic information. These properties make them particularly effective for traffic-sign recognition, lane-marking interpretation, object classification, and the identification of contextual attributes that cannot be derived directly from sparse geometric measurements. However, camera performance depends strongly on illumination, contrast, optical cleanliness, exposure control, and the representativeness of the training distribution. LiDAR provides direct three-dimensional range information and supports accurate geometric reconstruction of objects, road surfaces, and free space. Its measurements are less dependent on visible texture, although they remain sensitive to reflectivity, precipitation, atmospheric particles, window contamination, and the scanning characteristics of the device. Automotive radar directly measures range and radial velocity and generally provides greater resilience to darkness, fog, and moderate precipitation. At the same time, conventional radar measurements may offer lower angular resolution and more ambiguous object representations than camera or high-resolution LiDAR data. Inertial sensors, wheel odometry, GNSS receivers, ultrasonic devices, thermal cameras, and digital maps provide additional information for localization, near-field perception, low-light operation, and motion estimation.

The value of this modality diversity depends not only on the number of installed sensors but also on whether their physical principles generate genuinely complementary evidence. Multisensor fusion can reduce ambiguity when different modalities observe the same object through independent or partially independent mechanisms. Camera semantics can support the interpretation of geometrically sparse radar detections, while radar velocity measurements can stabilize motion estimation when visual appearance is unreliable. LiDAR geometry can improve depth estimation and object boundaries, whereas thermal imagery may preserve pedestrian visibility when visible-light contrast is insufficient. Nevertheless, complementarity should not be equated automatically with redundancy. A system may contain several modalities but still depend critically on one preferred representation, one calibration chain, one central processor, or one shared power and communication infrastructure. The resulting architecture may appear redundant at the hardware level while remaining vulnerable to common-cause failures at the system level [8].

Sensor fusion also creates additional technical dependencies that are absent from isolated modality evaluations. Measurements must be transformed into a common spatial reference frame, temporally aligned, associated with physical objects, and integrated while accounting for uncertainty. Extrinsic calibration errors can create systematic spatial displacement between modalities, while inaccurate intrinsic calibration can distort the measurements produced by an individual sensor. Timestamp differences may cause moving objects to appear at inconsistent locations, particularly at high relative velocities or during rapid ego motion. Communication delays, buffering, packet loss, and different sensor frame rates can further reduce temporal coherence. Fusion algorithms must therefore determine not only what each sensor observes but also when the observation was valid and how reliable it remains at the moment of decision. If these dependencies are ignored, adding sensors can increase the quantity of data without increasing the reliability of the resulting world model.

The development and evaluation of autonomous-vehicle sensing have been strongly influenced by public datasets and shared benchmarks. KITTI established one of the earliest widely adopted frameworks for synchronized camera and LiDAR evaluation in object detection, tracking, stereo vision, visual odometry, and scene understanding [9,10]. Its influence extended beyond the individual tasks because it provided common coordinate systems, annotations, evaluation procedures, and baseline methods. However, KITTI also reflects a specific sensor configuration, limited geographic coverage, particular traffic conditions, and a hardware generation that differs from contemporary production-oriented sensing systems. Its continued use is scientifically valuable, but results obtained on KITTI should not be interpreted as direct evidence of robustness across modern ODDs.

Later multimodal datasets substantially expanded the scale and complexity of available evidence. The nuScenes dataset introduced a broad sensor suite containing cameras, LiDAR, radar, GNSS, and inertial measurements together with temporally linked annotations and map information [11]. The Waymo Open Dataset increased the number and diversity of driving sequences and provided dense annotations for large-scale detection and motion-related research [12]. Argoverse and Argoverse 2 placed additional emphasis on detailed maps, forecasting, tracking, and geographically diverse urban scenes [13]. PandaSet contributed synchronized multimodal data collected using a sensor configuration that included high-resolution LiDAR and multiple cameras. Collectively, these resources enabled more reproducible comparisons of detection, tracking, forecasting, and fusion algorithms. They also accelerated the development of bird’s-eye-view representations by providing the geometric and temporal information necessary to train models across multiple viewpoints.

Visual datasets broadened the study of scene diversity and long-term environmental variation. BDD100K provided large-scale driving videos with annotations spanning detection, lane marking, drivable areas, tracking, and other tasks [14]. The Oxford RobotCar dataset enabled repeated-route analysis under substantial seasonal, weather, illumination, and traffic variation [15]. Such datasets are particularly important for understanding distribution shift because they document how the same or similar locations may appear under different environmental conditions. Nevertheless, visual diversity should not be confused with complete ODD coverage. Dataset composition is influenced by collection routes, vehicle operating schedules, local climate, annotation budgets, legal restrictions, and the practical limitations of sensor platforms.

Radar-focused and adverse-condition datasets addressed limitations that were underrepresented in conventional camera–LiDAR benchmarks. The Oxford Radar RobotCar dataset supported research on radar odometry and localization using repeated traversals under varying conditions [16]. RADIATE introduced radar-centric data for object detection and scene understanding in challenging weather and lighting conditions [17]. The DENSE and CADC datasets further supported the study of multimodal perception in snow, fog, and other difficult environments [18,19,20]. These resources have made it possible to compare radar, camera, LiDAR, and fusion performance under more demanding conditions. However, the resulting evidence remains heterogeneous. Weather categories may be based on qualitative labels rather than calibrated precipitation intensity, while sensor placement, target distance, road type, and annotation policy can vary substantially across datasets. A method that performs well in one adverse-weather benchmark cannot therefore be assumed to provide equivalent robustness in another environment.

Public datasets offer reproducibility, but they also embed assumptions about sensing architecture. Each dataset reflects particular sensor models, fields of view, mounting heights, scan patterns, exposure settings, radar frequencies, preprocessing pipelines, and synchronization methods. An algorithm may learn characteristics that are specific to those choices rather than general properties of the modality. For example, a LiDAR detector trained on one beam configuration may not transfer directly to a lower-resolution device, while a camera model may exploit color processing or lens properties that differ across platforms. Radar results are especially dependent on the form of the provided measurement. Consequently, benchmark performance should be interpreted as evidence for a defined sensor-processing configuration rather than as a universal statement about cameras, radar, LiDAR, or any other modality.

Automotive radar illustrates why measurement-level reporting is essential. Radar datasets and processing pipelines may expose sparse point detections, range-azimuth maps, range-Doppler maps, elevation-aware tensors, three-dimensional radar cubes, or partially processed high-definition representations [21,22]. These products retain different amounts of information about weak targets, multipath effects, elevation, micro-Doppler signatures, clutter, interference, and measurement uncertainty. Point-based outputs are computationally efficient and can be integrated readily with point-cloud or object-level pipelines. However, they may be produced by proprietary detection and filtering stages that remove information available in the underlying signal. Tensor-level or complex-valued measurements preserve richer evidence but require substantially greater storage, processing, and model complexity.

Recent radar detection studies demonstrate that the selected representation directly influences the observability available to the learning algorithm [23,24,25]. A model operating on highly filtered point targets cannot be assumed to exploit the same information as a model trained on range–Doppler or range–azimuth tensors. The distinction is also important for safety analysis. Proprietary preprocessing can reduce false alarms under nominal conditions, but it may simultaneously conceal why a target disappeared, why clutter increased, or how interference altered the signal. When only the final point detections are available, it becomes difficult to determine whether a failure originated in the physical channel, the signal-processing stage, the object detector, or the fusion architecture. Safety-oriented reporting should therefore identify the radar processing level, thresholding procedures, filtering assumptions, and uncertainty information available to downstream components.

Specialized datasets reveal further sensing trade-offs that are not captured fully by standard multimodal leaderboards. SemanticKITTI extends sequential LiDAR data with point-wise semantic labels and supports the study of temporal point-cloud understanding [26]. Cityscapes provides high-quality pixel-level annotations for urban visual scenes and remains influential in semantic segmentation and scene interpretation [27]. ACDC adds adverse-condition imagery and corresponding annotations across fog, night, rain, and snow, thereby enabling more targeted analysis of visual domain shift [28]. These datasets make it possible to isolate particular perception problems, but they address different spatial scales, tasks, annotation regimes, and sensor configurations. Their results should therefore be synthesized carefully rather than combined into a single ranking of sensor quality.

Multispectral and thermal benchmarks provide evidence that visible-light imaging is not the only relevant optical modality. Thermal cameras detect emitted infrared radiation rather than reflected visible light and may therefore retain pedestrian contrast under darkness or low visible illumination. Multispectral pedestrian datasets have demonstrated that thermal and visible channels can provide complementary information, particularly when one channel is degraded [29,30]. Yet thermal sensing also has limitations related to lower spatial resolution, reduced texture, temperature-dependent contrast, environmental reflections, and the thermal behavior of objects. Thermal cameras should consequently be treated as additional evidence channels rather than universal substitutes for visible-light imaging.

Event cameras represent another alternative sensing principle. Instead of recording complete image frames at a fixed rate, event sensors asynchronously report changes in brightness at individual pixels. This architecture provides high temporal resolution, low latency, and high dynamic range, which may be valuable during rapid motion, abrupt illumination changes, or high-contrast scenes. DSEC and related event-vision studies have expanded the empirical basis for event-based stereo, optical flow, depth estimation, and driving-scene perception [31,32]. Nevertheless, event sensing remains less mature in automotive datasets and production pipelines than conventional frame-based imaging. Event streams also require specialized representations, calibration procedures, and learning architectures. Their performance should therefore be evaluated in relation to specific failure modes rather than presented as a general replacement for cameras.

The diversity of available datasets highlights a broader methodological problem: evidence from different sensing studies cannot be collapsed into a universal hierarchy of “best” sensors. A semantic-segmentation dataset evaluates different capabilities from a long-range detection dataset. A pedestrian benchmark does not necessarily reveal localization performance, and a radar odometry dataset does not measure traffic-sign recognition. Similarly, datasets collected in natural snow cannot be compared directly with controlled fog-chamber experiments unless visibility, target characteristics, sensor configuration, and evaluation metrics are aligned. Meaningful synthesis requires the task, sensing level, ODD conditions, target range, measurement representation, and evaluation protocol to be reported together.

An additional limitation is that most datasets focus on environmental difficulty rather than explicit sensor health. A difficult scene and a degraded sensor can produce similar perception symptoms but require different system responses. Low camera confidence may result from an intrinsically ambiguous scene, heavy rain, a contaminated lens, overexposure, a damaged optical element, or a failed image-processing pipeline. Similarly, sparse LiDAR returns may be caused by low-reflectivity material, a long target distance, precipitation, partial aperture blockage, or hardware degradation. Without sensor-health labels or diagnostic metadata, an algorithm may detect reduced perception confidence without identifying the physical source of the problem. This distinction is essential because the appropriate mitigation depends on whether the environment is inherently difficult or the sensing channel itself is malfunctioning.

Complementarity therefore does not automatically produce fault tolerance. A fused system may fail when one modality is structurally privileged by the network architecture, when calibration drift remains undetected, or when a corrupted stream contributes confident but incorrect features. Early fusion can preserve detailed cross-modal interactions, but it may also allow one degraded input to contaminate the shared representation. Feature-level fusion can learn strong relationships between modalities, although these relationships may become unreliable when a modality is missing or shifted outside its training distribution. Decision-level fusion provides more modular separation, but it may discard useful low-level evidence and inherit overconfidence from independently trained detectors. No fusion level is intrinsically safe; robustness depends on uncertainty estimation, missing-modality handling, diagnostic coverage, and the independence of the contributing channels.

Modern bird’s-eye-view and transformer-based fusion architectures have achieved substantial improvements on multimodal perception benchmarks. These methods transform camera, LiDAR, or radar information into a common spatial representation and use attention mechanisms or learned associations to integrate evidence across views and time. However, benchmark accuracy under clean and synchronized input does not reveal how confidence behaves under sensor blockage, timestamp error, saturation, dropped frames, packet loss, or gradual contamination. A fusion model may retain plausible outputs even when one input has become physically unreliable, particularly if the training objective rewards average detection performance rather than explicit failure recognition. This creates a risk that the downstream planner receives a confident world model whose uncertainty does not reflect the actual state of the sensing system.

Environmental degradation affects different modalities through different mechanisms. Camera performance may deteriorate because of glare, low illumination, headlight bloom, motion blur, water droplets, mud, snow, or optical occlusion. LiDAR can experience attenuation, backscatter, ghost returns, reduced effective range, or partial field-of-view loss. Radar may be affected by mutual interference, multipath propagation, clutter, saturation, or ambiguous detections. GNSS can become unavailable in tunnels and urban canyons and may also be exposed to jamming or spoofing. Inertial sensors remain locally available but accumulate bias and drift when external corrections are absent. These modality-specific degradation mechanisms have been examined across adverse-weather, interference, calibration, and sensor-failure studies [33,34,35].

A safety-relevant architecture must do more than preserve average perception accuracy under such conditions. It must detect that degradation is occurring, determine which sensing functions are affected, estimate whether the remaining evidence is sufficient for the current driving task, and adapt vehicle behavior accordingly. For example, the loss of long-range camera visibility may be tolerable at low speed in a structured environment if radar and LiDAR remain reliable, but it may be unacceptable for high-speed automated driving where early recognition of distant hazards is required. The consequences of a sensor fault therefore depend on the ODD, vehicle speed, road geometry, maneuver, traffic density, and availability of alternative evidence. Sensor health should be interpreted in functional terms rather than as a binary hardware status.

Calibration and synchronization failures are especially important because they can generate coherent but incorrect measurements. A fully blocked camera may be detected relatively easily, whereas a small extrinsic-calibration drift may produce plausible observations that are displaced systematically. In multisensor systems, such displacement can cause incorrect object association, duplicated tracks, distorted object dimensions, or inconsistent free-space boundaries. Timestamp errors can produce similar effects for moving objects even when spatial calibration is correct. Gradual faults are particularly challenging because the system may continue to operate while performance deteriorates incrementally. Research on calibration monitoring and online consistency checking is therefore central to the safe deployment of multimodal perception [36,37,38].

Cybersecurity further extends the sensing problem beyond accidental degradation. GNSS spoofing, sensor replay, manipulated camera input, LiDAR interference, radar interference, and corrupted communication channels can alter the evidence available to the automated-driving system. An attack may be designed to resemble a natural sensor failure, making diagnosis more difficult. Conversely, an overly sensitive security mechanism may interpret unusual but legitimate environmental conditions as malicious activity. Robust sensing architectures must therefore combine physical plausibility checks, temporal consistency, cross-modal verification, and secure communication rather than relying solely on conventional object-detection confidence [39,40,41].

Graceful degradation requires explicit links between perception quality and operational decision-making. Once degradation is detected, the system must determine whether it can continue the current function, operate under reduced capability, request human intervention where applicable, execute a fallback maneuver, or transition to a minimum-risk condition. The appropriate response cannot be determined solely from the presence or absence of one sensor. It depends on whether the remaining sensing configuration satisfies the performance requirements of the current driving task. A blocked rear camera may have limited relevance during straight motorway travel but become critical during a lane change or reverse maneuver. Similarly, reduced localization accuracy may be tolerable on a low-speed open road but unacceptable near complex intersections or road boundaries.

This functional interpretation is consistent with the need to integrate perception engineering with functional safety, safety of the intended functionality, and cybersecurity. Functional-safety processes primarily address hazards caused by malfunctioning behavior, whereas SOTIF considers hazards arising from functional insufficiencies and reasonably foreseeable misuse even when no conventional hardware fault is present. Perception systems are affected by both categories because inadequate environmental evidence may result from physical failure, algorithmic limitation, insufficient training coverage, or an environmental condition outside the validated capability. Cybersecurity adds deliberate manipulation to this set of causes. Although these areas are frequently studied in separate research communities, they share a dependence on observable sensor limitations, uncertainty, diagnostic evidence, and operational mitigation [42,43,44].

The fragmentation of these communities has consequences for the literature. Sensor reviews frequently compare nominal attributes such as range, resolution, cost, field of view, and weather sensitivity but provide less analysis of how raw-measurement degradation propagates into object tracks, occupancy estimates, localization, prediction, and planning. Fusion surveys often classify methods as early, intermediate, or late fusion without determining whether the architecture remains operational when a modality is missing, corrupted, delayed, or miscalibrated. Adverse-weather studies commonly report results obtained using different facilities, target materials, ranges, precipitation intensities, visibility levels, and metrics. This heterogeneity prevents direct claims that one sensor or fusion method is universally superior. Functional-safety and cybersecurity studies, meanwhile, may define hazards and attack surfaces without quantifying the perception consequences using the same metrics employed in computer-vision or robotics research.

A further gap concerns the distinction between perception performance and perception assurance. High detection accuracy is necessary, but it does not demonstrate that the system can identify the limits of its own evidence. A safety-critical sensing system should provide information about confidence, uncertainty, sensor availability, calibration validity, and the consistency of observations across time and modalities. Confidence scores produced by neural networks are not automatically equivalent to calibrated probabilities, and low confidence is not automatically diagnostic of sensor failure. Assurance therefore requires additional mechanisms, including out-of-distribution detection, residual analysis, cross-modal consistency checking, temporal plausibility tests, calibration monitoring, and independent safety channels.

The reviewed evidence also indicates that robustness must be evaluated at several levels. At the physical level, studies should characterize how weather, contamination, interference, temperature, vibration, and aging affect raw measurements. At the signal-processing level, they should report filtering, thresholding, compression, and proprietary preprocessing. At the perception level, evaluation should quantify detection, segmentation, tracking, occupancy, and localization performance. At the system level, research should establish whether the resulting errors alter planning, control, and minimum-risk behavior. A sensing architecture that loses several percentage points of average precision may remain operational if uncertainty is correctly recognized and vehicle behavior is adapted. Conversely, a small but systematic localization bias may create severe risk if it is undetected and aligned with a critical road boundary.

Accordingly, this review addresses four interconnected gaps. First, existing sensor reviews often compare nominal modality characteristics while giving insufficient attention to the propagation of physical and algorithmic failures from the raw measurement level to the world model. Second, fusion surveys typically organize methods according to computational architecture but rarely evaluate whether the resulting systems remain dependable under missing, corrupted, delayed, or overconfident modalities. Third, adverse-weather and degradation studies are evaluated using heterogeneous experimental facilities, targets, ranges, environmental intensities, and metrics, limiting the validity of cross-study rankings. Fourth, perception, functional safety, SOTIF, and cybersecurity remain partially separated in the literature despite their common dependence on measurable sensing limitations and effective operational responses.

The objective of this review is therefore to synthesize autonomous-vehicle sensing research through the lens of safety-critical robustness rather than nominal benchmark accuracy alone. The analysis considers the physical properties of the principal sensing modalities, the representations used by fusion architectures, the datasets and experimental procedures used for evaluation, and the mechanisms through which uncertainty and degradation are communicated to the wider automated-driving system. Particular attention is paid to whether studies evaluate clean-data performance only or also address contamination, weather, calibration drift, interference, spoofing, sensor loss, and common-cause dependencies.

Five research questions structure the review. RQ1 asks which functional capabilities, physical limitations, and failure modes characterize the principal sensing modalities used in automated vehicles. RQ2 examines which fusion representations and architectural strategies improve perception and localization and identifies the dependencies created by these approaches. RQ3 investigates how adverse weather, contamination, calibration drift, communication delay, interference, spoofing, and partial sensor loss are evaluated. RQ4 assesses the evidence supporting sensor-health monitoring, uncertainty-aware fusion, fault isolation, graceful degradation, and fallback behavior. RQ5 identifies the datasets, metrics, reporting practices, and validation procedures required to make safety-related claims reproducible and comparable.

**Figure 1:**
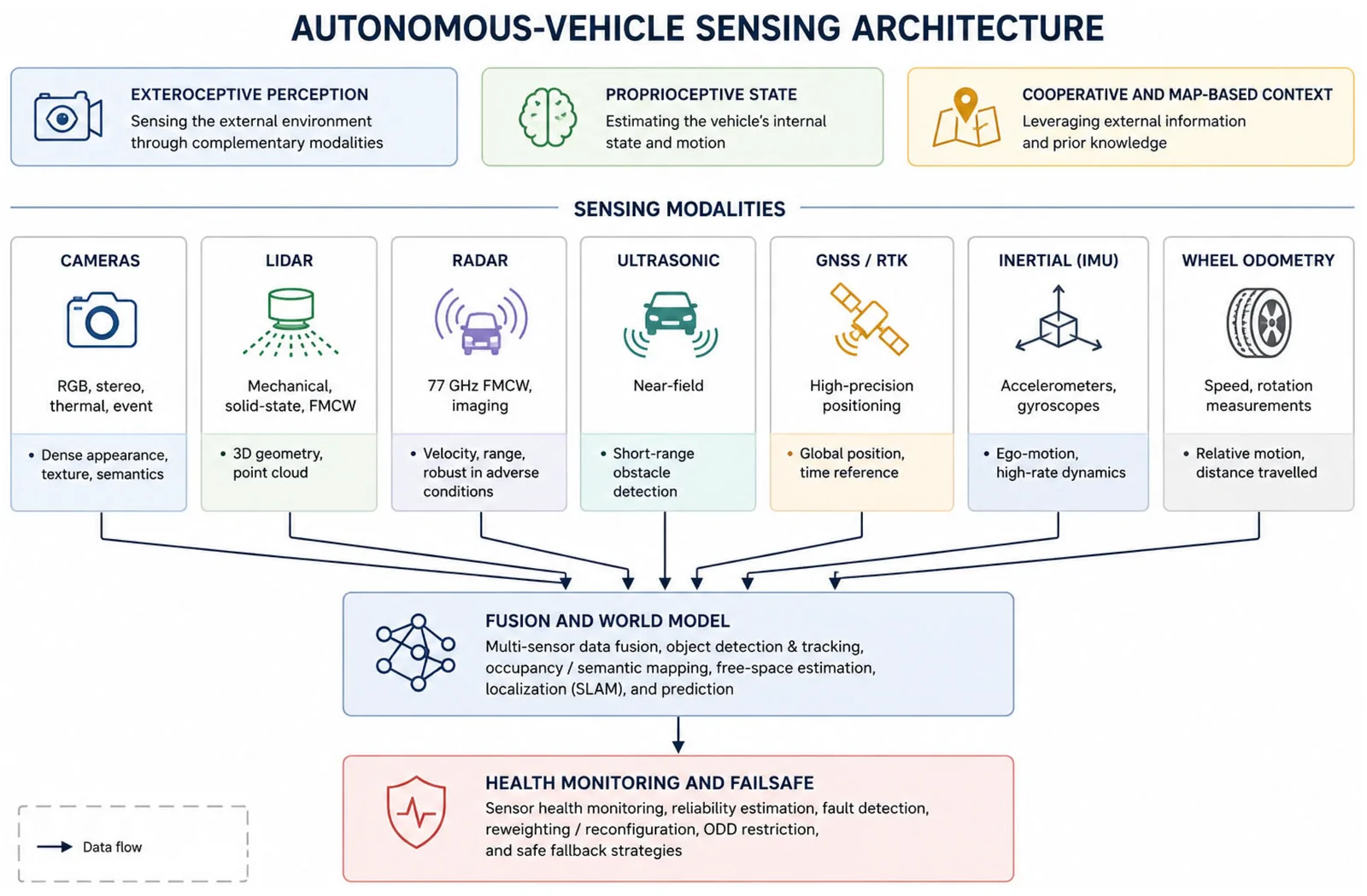
Taxonomy of autonomous-vehicle sensing, fusion, world modeling, and health-aware fallback. Conceptual synthesis based on functional-safety, SOTIF, sensor-fusion, adverse-weather, and fault-tolerance literature [6,7,8,18,33,34,35,36,37,38,39,40,41,42,43,44].

The contribution of the review is fourfold. First, it proposes a modality-to-safety taxonomy that links physical sensing principles to characteristic degradation mechanisms and downstream functional consequences. Second, it develops a structured evidence map covering sensors, fusion levels, datasets, environmental conditions, faults, attacks, health-monitoring mechanisms, and operational responses. Third, it provides a cross-dataset critique that distinguishes task-specific benchmark results from broader claims about modality robustness. Fourth, it presents an architectural perspective in which sensor-reliability estimates are connected explicitly to perception confidence, operational restrictions, degraded modes, fallback strategies, and minimum-risk behavior. Through this structure, the review aims to shift the evaluation of autonomous-vehicle sensors from isolated accuracy comparisons toward evidence-based assessment of whether an automated-driving system can recognize, tolerate, and safely respond to the limitations of its own sensing architecture. The resulting modality-to-safety taxonomy is summarized in Figure 1.

## 2. Materials and Methods

### 2.1. Protocol, Reporting Standard, and Review Scope

The review was reported in accordance with PRISMA 2020 and PRISMA-S [45,46]. The review was registered in PROSPERO on 30 July 2026 (CRD420261465869). The registration was retrospective because pilot work, formal searching, and screening had already been undertaken before the record was published. The eligibility criteria and synthesis plan were formalized during the review process, and subsequent protocol clarifications, including the final search end date and the separation of primary evidence from contextual sources, were documented in the review audit trail. No separate Open Science Framework registration was used. PRISMA was applied as a reporting and audit framework, not as a domain-specific risk-of-bias instrument.

The review uses a Population–Concept–Context logic adapted to engineering evidence synthesis. The population is road-going automated or autonomous vehicles and closely related perception platforms. The concept comprises onboard or cooperative sensing, calibration, synchronization, localization, sensor fusion, degradation, health monitoring, uncertainty, failure diagnosis, and mitigation. The context includes urban, suburban, highway, parking, adverse-weather, proving-ground, and simulation environments where the findings are explicitly connected to automated road-vehicle operation. The primary unit of analysis is a peer-reviewed study reporting a sensor method, dataset, experiment, quantitative evaluation, or safety-relevant architecture.

The review was guided by five questions: RQ1, What capabilities and failure modes characterize the principal sensing modalities? RQ2, Which representations and fusion architectures improve perception or localization, and what dependencies do they create? RQ3, How are adverse weather, contamination, interference, calibration drift, desynchronization, spoofing, and sensor loss evaluated? RQ4, What evidence supports sensor-health monitoring, uncertainty-aware fusion, graceful degradation, and fallback? RQ5, Which datasets, metrics, and reporting practices are needed to make safety claims reproducible? These questions were defined before the thematic synthesis and determined the extraction fields and table structure.

Automated-driving terminology follows SAE J3016, while the interpretation of safety evidence distinguishes malfunctioning behavior, functional insufficiency, and safety-case reasoning in line with ISO 26262, ISO 21448, and UL 4600 [1,33,34,35]. Product-specific performance claims were not treated as peer-reviewed evidence unless a reproducible experimental method and traceable source were available.

Terminology was fixed before coding. Sensor degradation denotes a partial or progressive loss of measurement quality relative to the specified nominal condition; sensor failure denotes loss or violation of a required sensor function; perception failure denotes a downstream task output that violates its acceptance requirement, irrespective of sensor cause; uncertainty denotes quantified lack of knowledge or variability in an estimate; and sensor health denotes the estimated condition and capability of a sensing channel, including hardware, timing, calibration, and environmental observability. These terms are not used interchangeably.

The protocol also distinguishes the technical object being evaluated from the level of claim made by the authors. A transducer-characterization study can support statements about signal attenuation or measurement noise, but it cannot by itself establish perception robustness. A perception experiment can support task-level conclusions within the tested data distribution, but it does not demonstrate a complete fallback strategy. A vehicle or proving-ground study can provide stronger system evidence, although its external validity still depends on scenario coverage and ODD definition. Coding this evidence level prevents results from different layers of the sensing chain from being treated as interchangeable and provides a consistent basis for the certainty interpretation used in the Discussion [1,33,34,35,45,46]. No physical analytical equipment was used in this literature-based review. No specialized statistical-analysis software was used; descriptive counts were calculated directly from the completed screening and extraction matrices and checked against the retained audit log.

### 2.2. Eligibility Criteria

Eligibility was defined at both report and study levels. A report was eligible when it contained sufficient methodological detail to identify the sensor configuration, task, evaluation setting, and principal outcome. Multiple reports describing the same experimental study were linked and treated as one evidence unit where appropriate, while genuinely distinct experiments from the same research group were retained separately. This distinction prevents double counting of dataset introductions, conference-to-journal extensions, and repeated benchmark results. Foundational standards, methodological reviews, and dataset papers were retained as contextual sources, but only peer-reviewed primary studies entered the modality and failure-mode evidence matrix.

The scope deliberately included both perception and localization because vehicle-level safety depends on their interaction. Studies limited to component physics were included only when the findings were explicitly connected to road-vehicle sensing, such as attenuation, interference, contamination, calibration, or measurement uncertainty. Purely algorithmic papers were excluded when the sensor assumptions could not be reconstructed or when evaluation relied solely on synthetic inputs without a defensible link to a road-driving sensor. Simulation studies were eligible when the simulator, fault model, parameter ranges, and validation basis were reported, because controlled corruption and failure injection are often impractical or unsafe to reproduce on public roads [1,45,46].

Eligibility decisions also considered the relationship between the reported outcome and the review questions. Studies were not excluded merely because their principal objective was accuracy rather than reliability; they were retained when the sensor assumptions, representation, or ablation results informed the analysis of observability and failure dependence. In contrast, papers that used autonomous-driving terminology only as an application example were excluded if the experiment could not be connected to a road-vehicle sensor configuration. This rule maintained a broad coverage of enabling methods while preventing the evidence map from becoming a general review of computer vision, robotics, or machine learning. Table 1 summarizes the eligibility criteria applied during screening.

### 2.3. Information Sources, Search Dates, and Search Strategy

The final searches were executed on 29 July 2026 in Scopus, Web of Science Core Collection, IEEE Xplore, and ScienceDirect. The eligibility period was January 2015 to 29 July 2026, with earlier foundational publications retained only when required to explain a dataset, calibration method, localization method, or safety standard. Searches were restricted to English-language articles and conference/proceedings papers and to title, abstract, and keywords where supported. Google Scholar was used only for citation checking and locating accessible versions; it was not counted as a primary database.

The executed search combined an automated-driving block with sensor-modality and perception/reliability blocks. The conceptual blocks are summarized in Table 2, and Table 3 reports the exact strings, platform fields, filters, execution date, and returned counts: Scopus 712, Web of Science 604, IEEE Xplore 458, and ScienceDirect 369 records (database total *n* = 2143). Export files were retained in RIS or BibTeX format with database name, execution date, and returned count in the audit log.

Search sensitivity was prioritized because reliability terminology is fragmented across perception, robotics, functional safety, and cybersecurity. Backward and forward citation checking was used to test coverage and identify contextual standards or reviews; it did not add a primary-study record to the final PRISMA identification total.

### 2.4. Record Management and Deduplication

All database exports were saved in RIS or BibTeX and merged into a version-controlled reference library. DOI strings were normalized by lowercasing, removing URL prefixes, and trimming punctuation. Exact duplicates were identified by DOI or normalized title, year, and first author; near-duplicates were manually checked to link conference and journal versions without double counting.

The combined identification set contained 2143 records. Before title/abstract screening, 612 duplicates, 86 records marked ineligible by predefined automation rules, and 95 records removed for other documented reasons were excluded (total removed *n* = 793), leaving 1350 records for screening. The automation rules used only objective bibliographic fields: language, publication year, and document type. All 86 flagged records were placed in a separate verification queue and manually checked by the author against the title, abstract, year, language, publication type, and source identifier before removal; any ambiguous record was returned to title/abstract screening. The audit log retained the source identifier, triggered rule, and manual confirmation for every automated removal. The 90 references in the bibliography comprise 65 included primary studies plus 25 contextual standards, reviews, and methodological sources; they are not a separate screening corpus.

For multiple versions of the same empirical study, the most complete peer-reviewed report was retained and related reports were linked. Distinct model variants were not merged solely because they shared a dataset or title stem. This rule produced one row per included primary study in Appendix A and prevented the same empirical result from entering the synthesis more than once. Table 4 details the record-management and deduplication workflow.

### 2.5. Study Selection, Full-Text Assessment, and PRISMA Flow

**Figure 2:**
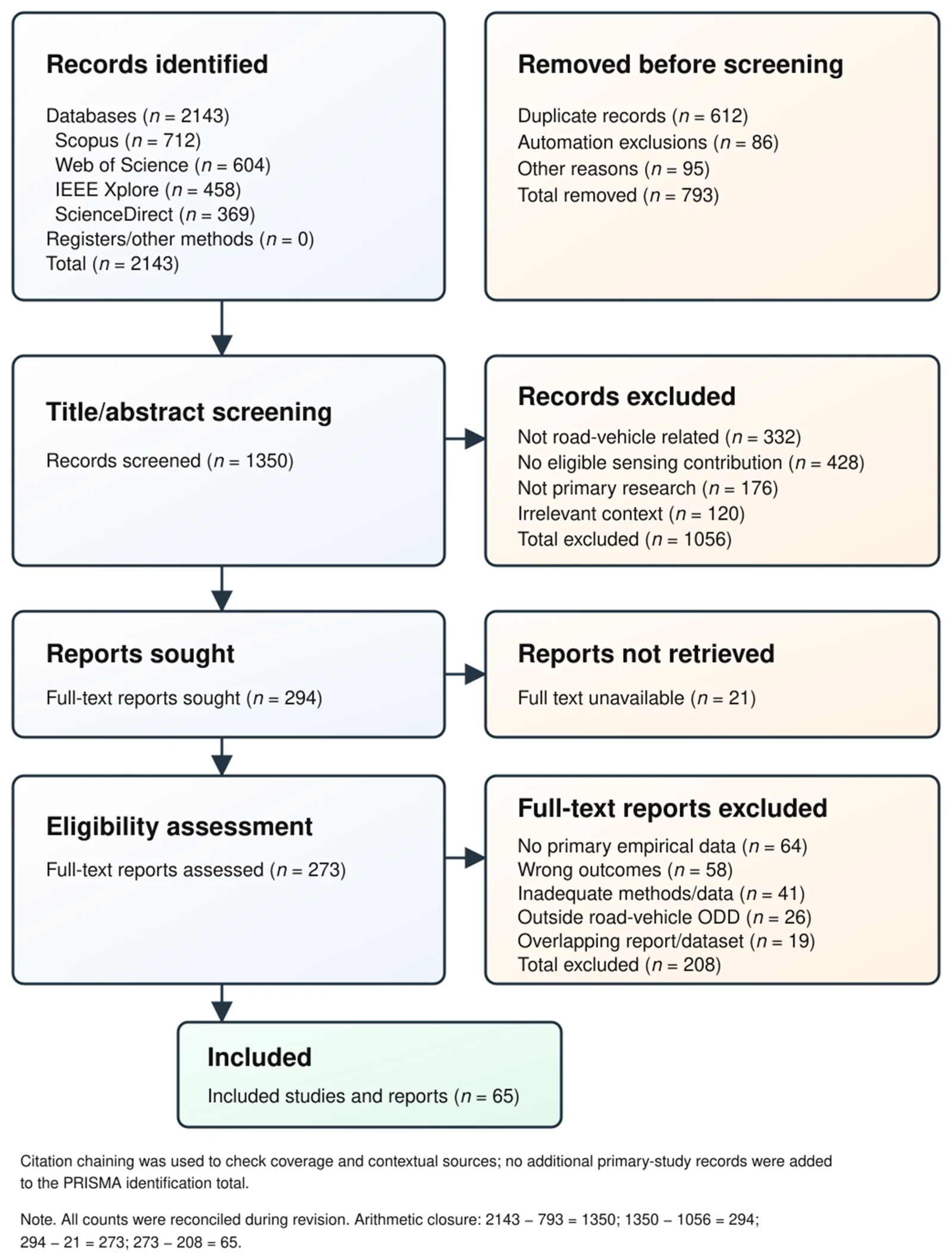
Final PRISMA 2020 flow diagram. Database identification, pre-screening removals, screening exclusions, non-retrievals, full-text exclusions, and the 65 included primary studies are reconciled across Section 2 and Section 3 and Appendix A [45,46].

The earlier draft incorrectly stated that two reviewers had independently screened all records. This single-author review used a two-pass procedure: title/abstract and full-text decisions were made once and then rechecked after a separate interval against the prespecified form, with reasons corrected in the audit log where necessary. Because the two passes were performed by the same reviewer and are not statistically independent, Cohen’s kappa and inter-reviewer raw agreement are not applicable and are not reported. This limitation is stated explicitly in Section 11. Figure 2 presents the reconciled PRISMA 2020 flow for this process.

Of 1350 screened records, 1056 were excluded at the title/abstract stage: not related to road vehicles (*n* = 332), no eligible sensing/fusion/localization/degradation contribution (*n* = 428), not primary research (*n* = 176), or irrelevant context (*n* = 120). Of 294 reports sought, 21 were not retrieved and 273 were assessed in full. Full-text exclusions were no primary empirical data (*n* = 64), wrong outcomes (*n* = 58), inadequate data or methods (*n* = 41), outside the road-vehicle ODD/domain (*n* = 26), and overlap with an included report or dataset (*n* = 19). The remaining 65 primary studies entered the synthesis and Appendix A.

The final PRISMA flow is arithmetically closed: 2143 identified minus 793 removed equals 1350 screened; 1350 minus 1056 excluded equals 294 reports sought; 294 minus 21 not retrieved equals 273 assessed; and 273 minus 208 full-text exclusions equals 65 included studies. Contextual references were not counted as included primary evidence.

Screening decisions retained a short justification field and one mutually exclusive final reason. Ambiguities involving generic robotics, sensor-design papers without road-vehicle validation, clean-only accuracy studies, and conference-to-journal extensions were resolved using the eligibility rules in Table 1.

### 2.6. Data Extraction and Coding

A structured extraction form records bibliographic information, sensor modalities, hardware and sampling assumptions, perception or localization task, dataset, environmental conditions, fusion stage, coordinate representation, calibration procedure, temporal aggregation, failure or corruption model, uncertainty method, evaluation metrics, real-time evidence, comparison baseline, code or data availability, and principal limitation. For dataset papers, the extraction additionally records geographic diversity, weather coverage, synchronization, sensor suite, annotation type, sequence duration, and whether sensor failures or sensor-state labels are represented.

The author performed the initial extraction and then verified every field in a separate full-text pass against tables, figures, and Supplementary Material. Numeric values were retained in their original units; missing information was coded as not reported rather than inferred. This repeat verification reduces transcription error but is not equivalent to independent duplicate extraction.

The coding framework separates sensor failure from perception failure. Sensor-state variables describe physical or communication degradation, such as blockage, attenuation, interference, timing offset, or bias. Task-state variables describe the resulting effect on detection, tracking, occupancy, or localization. This distinction prevents low task confidence from being misinterpreted as proof of sensor failure and supports the analysis of residual observability across modalities.

Extraction also distinguishes reported evidence from reviewer interpretation. Statements taken directly from an experiment, such as a measured detection decrease under a specified fog density, are coded separately from synthesis-level inferences about fallback or residual observability. For multimodal systems, the form records whether each modality has an independent encoder, whether features are projected into a common frame, whether modality masks or health variables are available, and whether training includes complete loss, partial degradation, or only nominal data. This granularity is required because two papers labeled “camera–LiDAR fusion” can expose very different failure behavior.

### 2.7. Methodological Quality Appraisal

A ten-item engineering-oriented methodological quality checklist was applied to all 65 included studies: sensor configuration; calibration/synchronization; dataset provenance; split integrity; baseline adequacy; metric validity; quantification of corruption severity when applicable; runtime evidence; uncertainty/variability; and reproducibility. Items were scored 0 (not reported), 0.5 (partially reported), or 1 (fully reported). The checklist qualified the interpretation of each study and was not used as an automatic exclusion threshold. The checklist and scoring rules are summarized in Table 5.

Quality appraisal is used to qualify the strength of conclusions rather than to generate an artificial pooled score. A study with excellent model reporting but a weak corruption model may still be informative for nominal perception, while it provides limited evidence about safety under degradation. Conversely, a small controlled experiment may offer strong causal evidence about a physical failure mechanism even if it does not demonstrate end-to-end driving performance. The synthesis therefore reports domain-level strengths and limitations and avoids treating all checklist items as equally important for every research question.

Particular attention is paid to leakage and comparability. Sequence data can produce optimistic results when adjacent frames from the same route appear in training and test sets, and synthetic corruptions can be tuned on the same benchmark used for evaluation. Studies receive stronger interpretation when splits are independent at the route or sequence level, baselines use the same input information and compute assumptions, and corruption parameters are fixed before testing. For adverse conditions, reporting a named dataset is not sufficient if the evaluated weather subset, visibility range, or sensor exposure is unclear. These checks do not imply that every paper must satisfy an identical experimental template; they identify which conclusions remain defensible under the available design.

The study-level appraisal was completed and rechecked during this revision for all 65 included studies and is provided as Supplementary File S1. Each item was verified against the included report and associated Supplementary Information. Explicit, complete reporting was scored 1; partial reporting or information available only indirectly through a named dataset or implementation was scored 0.5; and unreported information was scored 0 rather than inferred. Corruption severity was scored N/A for the 42 studies without an explicit degradation experiment. The domain totals in Table 6 were calculated directly from the 650 item-level judgements in Supplementary File S1.

### 2.8. Synthesis, Heterogeneity, and Certainty Interpretation

Because the literature spans object detection, segmentation, tracking, localization, odometry, calibration, adverse-weather sensing, and fault diagnosis, a single statistical meta-analysis is not appropriate across the full corpus. A structured narrative synthesis was therefore conducted. Evidence was grouped by physical sensing principle, task, fusion representation, degradation mechanism, diagnostic method, mitigation strategy, and operational consequence. Where multiple studies used the same benchmark and metric, results were compared qualitatively, but no pooled effect was calculated unless hardware, split, corruption severity, and evaluation protocol were sufficiently aligned. The synthesis prioritizes mechanisms and safety implications over isolated leaderboard values.

The synthesis follows a structured narrative-comparison process. Evidence is first grouped by modality and task, then by degradation mechanism, representation, and level of system integration. Within each group, conclusions are compared against the measurement physics, dataset composition, and evaluation design. Apparent disagreements are not resolved by vote counting; instead, the review examines whether they arise from different target materials, ranges, weather severity, preprocessing, sensor generation, or performance metric. This approach is more appropriate than meta-analysis when nominally similar studies do not estimate a common effect.

Cross-study tables were constructed to expose assumptions rather than to compress all results into a common score. For each modality, the synthesis relates the physical measurement principle to the observed failure mechanism, diagnostic indicator, downstream task effect, and possible mitigation. For fusion studies, the comparison records the point at which sensor information is combined and whether modality-specific uncertainty remains available. For datasets and benchmarks, the analysis emphasizes condition coverage, sensor configuration, annotation, and failure labels. This structure supports transparent qualitative synthesis even when numerical results cannot be pooled and allows readers to identify where the evidence is consistent, conditional, or absent.

Heterogeneity was assessed at five levels: hardware, data representation, task definition, environmental severity, and metric. Camera resolution or LiDAR channel count can change observability; radar point clouds are not equivalent to raw range–Doppler tensors; 3D detection metrics differ in class, range, and matching threshold; weather labels often omit visibility or precipitation intensity; and latency may refer only to network inference rather than the full sensing path. Claims of superiority were therefore restricted to comparable settings.

Certainty was interpreted through methodological quality, replication across datasets, realism of degradation, and consistency of the safety mechanism. Evidence was considered stronger when a result was reproduced across public and private data, tested under controlled and real adverse conditions, supported by calibrated uncertainty, and linked to an explicit operational response. Evidence from clean benchmarks, synthetic corruptions without validation, or single-sensor dropout was treated as informative but insufficient for vehicle-level safety claims.

## 3. Results

### 3.1. Study Selection and Evidence Profile

The final search identified 2143 records, of which 793 were removed before screening. After 1350 titles/abstracts and 273 full texts were assessed, 65 peer-reviewed primary studies were included. The bibliography contains 25 additional contextual references (standards, reporting guidance, surveys, and foundational sources) that informed definitions or interpretation but did not enter the primary-study evidence matrix. Appendix A contains exactly one row for each of the 65 included studies.

The evidence profile was uneven across the sensing stack. Camera and LiDAR studies dominated object detection, segmentation, and fusion, whereas radar studies were fewer and used less standardized measurement products. Localization research provided mature estimator architectures but often reported route-level accuracy without explicit integrity or failure-detection analysis. Adverse-weather datasets increased condition diversity, yet only a minority of studies represented contamination, intermittent loss, calibration drift, or timebase errors as labeled sensor states. This imbalance matters because robustness to naturally difficult scenes is not equivalent to diagnosis of an impaired sensor.

A second pattern was the separation between component-level and vehicle-level evidence. Many studies quantified changes in detection metrics, point density, signal strength, or localization error, but fewer propagated those changes to maneuver availability, stopping-distance requirements, or minimum-risk behavior. The review therefore treats a statistically significant benchmark improvement as an algorithmic result unless the study also demonstrates calibrated uncertainty, diagnostic performance, residual observability, or an operational response. This distinction prevents the synthesis from overstating the safety meaning of perception accuracy.

The corpus also showed a concentration of evidence around successful detection architectures. Fewer studies reported negative results, health-monitor false alarms, recovery after a transient fault, or interactions between simultaneous degradations. Complete sensor removal was common as an ablation because it is easy to reproduce, but abrupt removal does not represent many field failures, which may develop gradually and affect only a spatial sector, frequency band, or subset of channels. The synthesis therefore gives greater interpretive weight to studies that quantify severity, preserve the temporal development of the fault, and compare the monitor response with the actual change in task performance [36,37,38,39,40,41,42,43].

The evidence profile is strongly shaped by public benchmark availability. Camera and LiDAR studies dominate because KITTI, nuScenes, Waymo Open Dataset, Argoverse, BDD100K, and SemanticKITTI provide established annotations and metrics [9,10,11,12,13,14,26,27]. Radar evidence is more heterogeneous because datasets expose different processing levels, from target lists and point clouds to range–azimuth–Doppler tensors [16,17,21,22,23,24,25]. Thermal and event-camera studies remain specialized, while ultrasonic evidence is limited by the scarcity of open production data [29,30,31,32]. The technical corpus and contextual basis also cover driving simulators and LiDAR simulation [47,48,49], point-cloud detection architectures [50,51,52,53,54,55,56,57], camera–LiDAR fusion methods [58,59,60,61,62,63,64], radar–camera fusion systems [65,66,67,68,69], camera-only BEV methods [70,71,72,73,74,75,76], additional multimodal fusion methods [77,78], radar fusion and odometry studies [79,80,81], and LiDAR/visual–inertial localization and calibration methods [82,83,84,85,86,87,88,89,90].

### 3.2. Distribution by Sensor Modality, Task, and Reporting Practice

The final evidence map was dominated by camera (*n* = 45) and LiDAR (*n* = 35) studies; radar appeared in 18 and GNSS/IMU or inertial sensing in 10 studies. Thermal, gated, and event sensing appeared in four studies, while no eligible ultrasonic primary study was identified. Because multimodal papers contribute to more than one modality, these modality counts are non-exclusive.

By evidence level, 48 studies provided component or benchmark evidence, 12 provided controlled degradation or diagnostic evidence, and only 5 connected sensing results to an operational or system response. Accordingly, the review treats health-conditioned reweighting, ODD restriction, and minimum-risk behavior as synthesis-level design recommendations unless a cited study directly demonstrated the relevant link.

The completed appraisal showed the strongest reporting for metric validity (65/65 fully reported), baseline adequacy (62/65), dataset provenance (59/65), split integrity (57/65), and reproducibility (51/65). Sensor-configuration and calibration/synchronization reporting were more often partial: only 31 and 32 studies, respectively, received a full score. Runtime evidence was fully reported in 15 studies and absent in 7, while uncertainty or variability was fully quantified in only 6 studies and not reported in 29. Among the 23 studies with an explicit degradation experiment, 9 fully parameterized corruption severity and 14 reported only partial or categorical severity. These results support the cautious interpretation of robustness and operational-safety claims used throughout the synthesis.

Comparative statements were therefore limited to the tested hardware, dataset, environment, range, and metric. No table or narrative comparison should be interpreted as a universal ranking of modalities or architectures. Where denominators, event counts, repeated runs, or confidence intervals were missing, the conclusion was coded as limited rather than generalized beyond the reported experiment. Table 7 provides the resulting functional comparison of sensing modalities.

### 3.3. Cameras: Semantic Richness with Environment-Dependent Reliability

Cameras remain the most information-dense and economically scalable exteroceptive sensors. A single RGB frame contains lane markings, traffic-light state, sign text, object appearance, road texture, and contextual cues that are difficult to obtain directly from range sensors. Multi-camera systems can provide near-360° coverage, while stereo geometry, structure from motion, monocular depth networks, and temporal aggregation estimate scene depth. Cityscapes, BDD100K, KITTI, nuScenes, Waymo Open Dataset, and Argoverse have accelerated vision research by providing large annotated corpora [9,10,11,12,13,14,27]. Camera-only bird’s-eye-view approaches such as Lift-Splat-Shoot, BEVFormer, DETR3D, PETR, PETRv2, BEVDet, and BEVDepth demonstrate that strong 3D scene representations can be learned from synchronized multi-view imagery [70,71,72,73,74,75,76].

The safety limitation is not that cameras are inaccurate in general, but that their observability changes sharply. Low illumination reduces the signal-to-noise ratio, direct sun produces flare and saturation, wet roads generate specular reflections, lens droplets alter local contrast, snow or mud can partially block the field of view, and motion blur suppresses high-frequency features. A network may still return confident detections under these conditions because discriminative confidence is not equivalent to sensor validity. Image corruption benchmarks expose sensitivity to noise, blur, weather overlays, and digital perturbations, but many generic corruptions do not reproduce the spatially structured effects of real optical contamination [28,38,39].

Stereo cameras add an independent geometric constraint, yet their performance depends on accurate rectification, sufficient texture, non-repetitive patterns, and stable exposure. Depth uncertainty increases with distance and small disparity. Monocular depth can provide dense estimates but inherits training-domain bias and scale ambiguity. Multi-frame methods improve stability but introduce dependence on ego-motion and object-motion compensation. Consequently, vision health monitoring should combine image-quality indicators, lens-obstruction segmentation, exposure statistics, blur estimation, temporal consistency, and task-level residuals rather than relying on a single network confidence.

Thermal cameras can reveal warm road users in darkness and reduce dependence on visible illumination. The KAIST multispectral benchmark demonstrated the value of aligned RGB–thermal imagery for pedestrian detection [29]. However, thermal contrast varies with the season, ambient temperature, clothing, vehicle heat, and surface emissivity. Thermal sensors also provide limited color and text information. Their best role is therefore complementary rather than substitutive: they can restore pedestrian evidence when RGB quality is low, while the fusion system must still account for alignment differences and temperature-dependent contrast.

Event cameras report asynchronous brightness changes with a microsecond-level temporal resolution and high dynamic range. DSEC and related datasets have made event-frame and event-LiDAR research more accessible [30,31,32]. Event sensors are promising for rapid motion, flicker, and high-contrast transitions, but they do not directly produce conventional texture and can be sparse in static scenes. Event representations also depend on temporal windows, motion, and contrast thresholds. For autonomous driving, the evidence supports their use as a specialized high-rate complement, especially for optical flow and motion boundaries, rather than a complete replacement for frame cameras.

Camera configuration is itself a safety variable. Narrow-field telephoto cameras improve long-range angular resolution but create stronger sensitivity to vibration and alignment; wide-angle and fisheye cameras increase near-field coverage but require distortion-aware calibration and may allocate fewer pixels per distant object. Stereo baselines trade compact packaging against depth precision, and rolling-shutter sensors can create motion-dependent geometry. A review of camera performance should therefore report the focal length, field of view, baseline, shutter type, exposure control, frame rate, bit depth, lens heating or cleaning, and overlap between views.

Health monitoring should operate at multiple levels. Signal-level indicators include saturation, underexposure, noise, hot pixels, blur, and dropped frames. Scene-level indicators include horizon consistency, lane continuity, optical-flow residuals, and disagreement between overlapping cameras. Task-level indicators include calibrated detection uncertainty, track continuity, and cross-modal residuals. A single softmax confidence cannot identify whether a confident error arose from domain shift, lens contamination, or an ambiguous scene.

The reliability of camera perception is also affected by the interaction between optics and learned representations. Automatic exposure, high-dynamic-range processing, image signal processing, compression, and lens correction can improve nominal appearance while hiding the physical origin of a degradation. For example, a network may remain confident on a locally sharpened but globally low-contrast frame, or temporal filtering may preserve a lane hypothesis after the markings are no longer observable. Health monitoring should therefore combine preprocessing diagnostics with task-level residuals and should retain access to selected raw or minimally processed indicators when the safety architecture depends on identifying glare, saturation, blur, or contamination [27,28,29,30,31,32,38,39]. Table 8 compares the principal camera families and their safety-relevant characteristics.

### 3.4. LiDAR: Metric Geometry, Sparse Semantics, and Weather Sensitivity

LiDAR provides direct range measurements and remains a central source of metric 3D geometry for autonomous driving. PointNet and PointNet++ established direct learning on unordered point sets, while VoxelNet, SECOND, PointPillars, PointRCNN, PV-RCNN, and CenterPoint progressively improved accuracy and computational efficiency for 3D detection [50,51,52,53,54,55,56,57]. LiDAR supports object detection, occupancy estimation, curb and road-edge extraction, mapping, and localization. Unlike monocular vision, it does not infer range from learned appearance alone, and its geometry is comparatively stable across day and night.

LiDAR performance nevertheless depends on emitted wavelength, pulse energy, receiver sensitivity, scanning pattern, beam divergence, target reflectivity, incidence angle, range, and atmospheric transmission. Point density decreases with distance, and small or dark objects may return few points. Glass, water, retroreflectors, and highly absorbent surfaces can produce missing or atypical returns. Mechanical scanning systems add moving parts, while solid-state and FMCW designs create different field-of-view, resolution, velocity, and interference trade-offs. These hardware differences complicate attempts to generalize benchmark findings across sensor generations.

Rain, fog, and snow can attenuate the outgoing and returning signal and introduce backscatter points. The Seeing Through Fog and the Canadian Adverse Driving Conditions dataset demonstrated that weather-aware multimodal datasets are essential for evaluating these effects [18,19]. Simulation studies for fog and snowfall enable controlled severity sweeps, but synthetic particle distributions and optical models must be validated against real sensor recordings [36,37]. A particularly important distinction is between graceful point-density reduction and structured corruption: a partially blocked aperture can remove a coherent angular sector, whereas fog may create distributed near-range clutter and reduced target returns.

LiDAR health monitoring can exploit return-rate statistics, intensity distributions, spatial occupancy, ring consistency, temporal scan overlap, self-motion residuals, and cross-modal disagreement. However, a low point count may be legitimate in open space, and an intensity shift may reflect target composition rather than contamination. Reliable diagnosis therefore requires context. A blocked front sector is safety-critical during highway driving but may be less relevant during a constrained reverse maneuver if rear sensing remains valid. Health estimates should be converted into task-specific observability rather than treated as global binary status.

LiDAR degradation is often spatially nonuniform. A contaminated window, damaged channel, or partial occluder may remove only a sector or subset of scan rings, leaving the global point count within a nominal range. Sector-level occupancy statistics, ring-wise return distributions, intensity trends, and consistency with ego-motion are therefore more informative than a single health scalar. The downstream consequence depends on where the loss occurs: reduced rear coverage may be tolerable during straight low-speed motion but unacceptable for a lane change, while loss near the road surface can compromise free-space estimation even when distant objects remain detectable [18,19,20,26,36,37].

LiDAR redundancy also deserves careful interpretation. Two LiDARs with overlapping fields of view may share the same weather and contamination mechanisms. Diverse redundancy can be stronger: radar may preserve range and velocity in precipitation, while cameras may retain semantics when LiDAR returns are sparse. The architecture should therefore represent both independence and common-cause failure. Physical separation, heating and cleaning systems, diverse wavelengths, and differing viewpoints can reduce some common-cause vulnerabilities, but they add cost, power, calibration complexity, and maintenance demand.

LiDAR architecture affects the failure model. Rotating multi-beam systems provide a broad coverage and established scan geometry but include moving assemblies and temporally distributed scans. MEMS and optical-phased-array designs can reduce moving mass but may have narrower fields of view or nonuniform sampling. Flash LiDAR captures a full field simultaneously at shorter ranges, while frequency-modulated continuous-wave LiDAR can in principle provide direct velocity and improved interference discrimination. Evidence should not generalize from one hardware class to another without reporting the wavelength, scan pattern, field of view, range, angular resolution, return policy, and interference handling. Table 9 summarizes these LiDAR technology classes and their evidence requirements.

Cleaning and thermal management are part of the sensing system rather than ancillary vehicle hardware. Hydrophobic coatings, air jets, wipers, heaters, and washer systems can restore observability, but they have activation delays, energy and fluid costs, and residual blind zones. Diagnostic thresholds should therefore account for the expected recovery time and for whether contamination is localized or global. A safe architecture may need to reduce speed before cleaning completes, even if the perception network continues producing detections.

### 3.5. Automotive Radar: Velocity-Rich Sensing Under Sparse and Ambiguous Geometry

Automotive radar measures range, radial velocity, and angle through frequency-modulated continuous-wave processing. Its ability to obtain Doppler velocity directly is a major advantage for tracking, collision assessment, and motion segmentation. Radar also operates in darkness and generally experiences lower atmospheric attenuation than visible-light and near-infrared sensors. The Oxford Radar RobotCar, RADIATE, View of Delft, CARRADA, RADIal, and Astyx-related datasets have broadened research beyond proprietary point detections toward raw or tensor representations [16,17,21,22,23,24,25].

The main difficulty is representation. Production radars often output sparsified target lists after proprietary filtering, while research systems may provide analog-to-digital samples, range–Doppler maps, range–azimuth maps, four-dimensional tensors, or point clouds. Information lost in target-list processing cannot be recovered by a downstream network. Conversely, raw radar tensors are large, hardware-specific, and sensitive to antenna configuration and signal-processing choices. Cross-dataset transfer is therefore weaker than the common label “radar” suggests.

Radar returns are affected by multipath, sidelobes, clutter, mutual interference, and ghost detections. Angular resolution can be much poorer than range resolution, causing difficulty in separating adjacent objects. Static infrastructure may generate strong reflections, while vulnerable road users can produce weak or complex signatures. Deep radar models such as RODNet and raw high-definition radar networks seek to exploit the temporal and Doppler structure rather than treating radar as sparse pseudo-LiDAR [25,66]. Camera–radar methods add semantics, and LiDAR–radar methods combine geometric density with velocity evidence [65,66,67,68,69,79].

Radar health monitoring should distinguish complete loss, degraded sensitivity, interference, misalignment, timing error, and environment-induced clutter. Basic diagnostics include received power, noise floor, target count, range-Doppler occupancy, temperature, synchronization residuals, and comparison with ego-motion or map predictions. Yet a high target count can indicate either rich traffic or interference, while a low target count can indicate an empty road. As with LiDAR, health estimation benefits from temporal and cross-modal context.

Imaging radar is narrowing the angular-resolution gap and may become increasingly important for adverse-weather and long-range perception. However, an improved resolution does not remove the need for uncertainty modeling. Radar can preserve an obstacle hypothesis when camera and LiDAR degrade, but multipath can also produce plausible false objects. A safety-oriented fusion system should represent radar evidence as probabilistic occupancy, velocity distributions, or track confidence rather than convert it prematurely into deterministic points.

Four-dimensional imaging radar increases the antenna aperture, channel count, and elevation resolution, but the term does not define a single data product. Some systems expose detected points with elevation and Doppler; others provide range–azimuth–elevation–Doppler tensors. Preprocessing, constant-false-alarm-rate thresholds, clustering, and proprietary filtering can change the information available to a learning model. Publications should therefore state the raw measurement level and every irreversible processing stage.

Mutual interference is a system-level concern as radar-equipped traffic density increases. Interference can raise the noise floor, create structured artifacts, or suppress weak targets. Detection requires monitoring spectral occupancy, noise statistics, and temporal consistency, while mitigation can include waveform diversity, time–frequency coordination, interference cancellation, and cross-modal confirmation. The safety response should depend on the affected range and azimuth sectors, not merely on a global radar fault flag.

Radar interpretation must additionally account for target-dependent observability. The radar cross-section changes with the material, orientation, aspect angle, and multipath geometry, so a low-amplitude return is not by itself evidence of hardware degradation. Diagnostic reasoning benefits from comparing the measured noise floor, waveform integrity, channel balance, and expected scene dynamics over time. Cross-modal confirmation is useful, but the fusion system should preserve the possibility that radar is the only modality observing through spray, darkness, or partial visual occlusion. The design challenge is therefore to distinguish legitimate modality disagreement from a corrupted radar stream without suppressing the very diversity that provides redundancy [16,17,21,22,23,24,25,65,66,67,68,79,80,81]. Table 10 compares the principal automotive-radar data representations.

### 3.6. Ultrasonic, GNSS/IMU, Wheel Odometry, and Cooperative Context

Ultrasonic sensors remain common in production vehicles because they are inexpensive and effective for low-speed, near-field obstacle detection. Their wide acoustic beam can detect objects that lie below the vertical field of view of some cameras or LiDARs. The limitations are equally clear: short range, weak angular resolution, cross-talk, temperature dependence, and sensitivity to surface orientation and acoustic absorption. Ultrasonic sensing should therefore be treated as a maneuver-specific layer for parking and close-proximity safety, not as a general road-perception modality.

GNSS provides a global coordinate reference, while RTK and correction services can deliver high precision under favorable visibility. Urban canyons, tunnels, foliage, multipath, jamming, and spoofing can make position error non-Gaussian and abruptly discontinuous. IMUs provide high-rate angular velocity and specific force but accumulate bias and drift. Wheel odometry adds vehicle-motion constraints but is affected by the tire radius, slip, and road surface. Reliable localization therefore depends on multisensor estimation, map matching, and consistency monitoring rather than any single source.

Visual–inertial systems such as OKVIS, VINS-Mono, OpenVINS, ORB-SLAM2, and ORB-SLAM3 demonstrate the strength of tightly coupled state estimation [83,84,85,86,87]. LiDAR–inertial systems such as LIO-SAM are robust in texture-poor environments but depend on point-cloud structure and calibration [82]. Radar odometry offers an alternative under darkness, rain, and dust, although dynamic-object filtering and multipath remain challenges [80,81]. The strongest localization architectures use innovation tests, integrity monitoring, map consistency, and multiple independent motion cues to identify when one source becomes unreliable.

Cooperative perception and V2X can extend sensing beyond the line of sight, reveal occluded vehicles, and share infrastructure observations. Yet cooperative data are not equivalent to direct onboard measurements. Communication latency, packet loss, clock offset, sender localization error, message authentication, and trust become part of the sensing chain. The receiving vehicle must preserve provenance and uncertainty and should not allow unverified remote objects to override strong local evidence without consistency checks.

Localization reliability should be expressed through integrity as well as average error. A filter may report low root-mean-square error over a route while still producing rare, hazardous jumps in an urban canyon. Innovation tests, protection levels, map residuals, redundant odometry, and hypothesis monitoring can identify inconsistency, but their thresholds must be tied to lane-level and maneuver-level requirements. The relevant question is whether the position uncertainty remains inside a safe bound for the current driving task.

Common-cause failures are especially important in localization. Vision and LiDAR can both fail in heavy obscuration; GNSS and V2X can share communication or spoofing vulnerabilities; and wheel odometry and inertial estimates can both be corrupted by severe slip and vibration. Diverse sensing improves resilience only when the estimator retains independent residuals and avoids overconfident covariance collapse.

Integrity monitoring should operate at several time scales. Fast checks can detect packet loss, impossible increments, timestamp anomalies, and sudden innovation spikes, while slower checks identify thermal bias, map aging, wheel-radius changes, or gradual extrinsic drift. A localization system that only reports covariance from its estimator may remain overconfident when the assumed measurement model is wrong. Independent protection levels, multi-hypothesis tracking, map-feature diversity, and cross-checks between inertial, visual, LiDAR, radar, and wheel-derived motion can provide stronger evidence about whether lane-level positioning remains trustworthy [80,81,82,83,84,85,86,87]. Table 11 summarizes the localization and cooperative-sensing failure mechanisms and integrity indicators.

## 4. Calibration, Synchronization, and Representation

### 4.1. Spatial Calibration

Multimodal fusion assumes that observations can be expressed in a common coordinate system. Camera intrinsics, lens distortion, LiDAR–camera extrinsics, radar alignment, IMU orientation, and vehicle-frame definitions therefore constitute safety-critical parameters. Calibration methods range from target-based procedures to mutual-information, geometric-feature, and self-calibration approaches [88,89,90]. Even small angular errors can produce large spatial displacement at a distance, causing an object to be associated with the wrong image region or lane.

Calibration should not be viewed as a one-time manufacturing step. Mechanical shock, thermal expansion, service replacement, sensor mount movement, or body deformation can alter extrinsics. Online calibration monitoring can use lane geometry, road planes, object correspondences, horizon consistency, and ego-motion. A safety case should specify acceptable calibration envelopes, detection latency, and the operational response when an estimate exceeds tolerance. Simply retraining a network with augmentation does not replace explicit geometric assurance.

The effect of calibration error is task-dependent and non-linear. A small yaw error may have little effect on nearby free-space estimation but can shift projected evidence by several meters at a long range. Pitch error can bias road-plane estimation and object height, while time-varying vibration can produce a distribution of misalignment rather than a fixed offset. Calibration requirements should therefore be derived from the geometry and range of the safety-critical task. Reporting only a mean reprojection error is insufficient unless the spatial distribution, target range, temperature, vehicle motion, and acceptance threshold are also stated.

Online calibration creates its own assurance problem because an estimator can converge to an incorrect but internally consistent solution. Reliable monitoring should separate detection of inconsistency from automatic parameter correction and should impose rate limits, plausibility bounds, and independent validation before updated extrinsics influence safety-critical fusion. When the evidence is insufficient, the safer response may be to disable a fusion path, rely on a less calibration-sensitive representation, or restrict the ODD rather than continuously adapting the geometry [88,89,90].

Calibration validation should therefore include deliberate perturbation around the acceptance boundary. Rather than demonstrating only convergence from a favorable initial condition, experiments should test several directions and magnitudes of error, dynamic motion, temperature change, weak scene structure, and recovery after temporary sensor loss. The monitor should be evaluated for both missed miscalibration and false rejection of a valid configuration. These results can be translated into task-specific limits, such as maximum projection displacement at the stopping horizon or maximum orientation error compatible with lane assignment, which are easier to connect to a safety requirement than an abstract calibration residual [88,89,90].

### 4.2. Temporal Synchronization

Sensors operate at different rates and may use different clocks, exposure times, scan patterns, and network paths. A rotating LiDAR does not capture the entire scene at one instant, a rolling-shutter camera exposes rows sequentially, radar integrates chirps over a frame, and GNSS/IMU measurements have different delays. Temporal misalignment is especially harmful for moving objects and high-speed ego-motion. It can appear as spatial calibration error even when extrinsics are correct.

Fusion systems should record hardware timestamps, clock source, interpolation method, motion compensation, buffering delay, and maximum age of each observation. Health monitoring should detect not only missing packets but also stale data, jitter, clock drift, and inconsistent sequence ordering. Real-time guarantees must be assessed end to end, because a highly accurate network is not useful if the evidence arrives after the planning deadline.

Timing error should be evaluated as a distribution rather than a single nominal latency. Clock drift, network contention, batching, inference queues, sensor exposure, scan assembly, and motion compensation all contribute to observation age. The same mean delay can produce different risk depending on jitter and the relative motion of the target. For a crossing pedestrian or cut-in vehicle, a short but unmodeled temporal offset may be more harmful than a longer fixed delay that is explicitly compensated. Experiments should therefore inject controlled offsets and jitter at the measurement and feature levels and report the point at which association, tracking, or planning becomes unstable.

Temporal integrity also requires provenance through recurrent and multi-frame models. A current object hypothesis may depend on several earlier frames even when the latest sensor message is healthy. If a modality becomes stale or corrupted, cached features and tracks can continue to influence the world model. The architecture should record the age and source of accumulated evidence, define expiry rules, and prevent a high-confidence historical representation from masking the loss of present observability.

The required synchronization tolerance is likewise task- and range-dependent. Slow ego-motion in a parking maneuver may tolerate offsets that would be unacceptable for motorway tracking, and a distant oncoming vehicle can accumulate large projection error during a modest timestamp mismatch. A useful evaluation reports relative velocity, object range, sensor rate, exposure or scan duration, and the exact location where the delay is introduced. It should also separate sensing latency from network inference and fusion latency, because compensating for one component does not correct an unknown delay elsewhere in the chain.

### 4.3. Fusion Levels and Coordinate Representations

Classical fusion distinguishes early, feature-level, and late fusion, but modern architectures blur these categories. MV3D, AVOD, Deep Continuous Fusion, PointPainting, TransFusion, BEVFusion, CenterFusion, the Multi-Modal Fusion Transformer (TransFuser), FusionPainting, DeepFusion, and RadarNet illustrate different strategies for combining image, LiDAR, and radar information [58,59,60,61,62,63,64,65,66,67,68,69,77,78,79]. Bird’s-eye-view representations are attractive because they align perception with road geometry and planning, but projection into BEV can introduce discretization, occlusion, and depth uncertainty. Cross-attention can learn flexible associations, yet the learned weights are not necessarily calibrated measures of sensor reliability. Table 12 compares the fusion levels, representations, and safety implications.

Fusion should preserve provenance. A world-model object should carry which sensors observed it, observation age, geometric uncertainty, class uncertainty, and whether evidence is direct or inferred. Without provenance, downstream planning cannot distinguish a well-supported pedestrian track from a hypothesis generated by one degraded camera. Reliability-aware fusion can use gating, mixture-of-experts, Bayesian updating, evidential methods, or learned modality dropout, but training must include realistic corruption patterns and deployment-time health indicators.

Fusion architectures can fail even when each sensor remains individually plausible. Projection errors can associate a LiDAR point with the wrong image feature; attention may amplify a corrupted modality; temporal aggregation may propagate stale evidence; and end-to-end training may hide whether performance depends on one privileged sensor. Ablation under complete sensor removal is necessary but not sufficient. Evaluation should include partial field-of-view loss, sector-specific noise, timing offsets, calibration drift, false returns, and correlated environmental degradation.

Representation choice determines which failures remain visible to the fusion module. Early fusion retains fine measurement detail but requires accurate alignment and can spread corruption through shared layers. Feature-level fusion offers flexibility, yet learned embeddings may hide whether a feature originated from one sensor or several. Late fusion preserves modularity and makes sensor-level diagnostics easier to apply, but it can lose complementary information before association. Hybrid architectures can combine these strengths when they maintain modality-specific pathways, explicit masks, uncertainty estimates, and a fallback output that does not depend on every branch [8,44,58,59,60,61,62,63,64,65,66,67,68,69,70,71,72,73,74,75,76,77,78,79].

A reliability-aware fusion model should be evaluated along a continuum from nominal operation to complete loss. Real failures are often gradual, local, and correlated: contrast falls over several seconds, a LiDAR sector becomes intermittently blocked, radar noise increases in a limited band, or timestamps drift under compute overload. Training only with whole-modality dropout may improve tolerance to missing input while leaving the system vulnerable to plausible but misleading features. Severity-controlled corruption, health-conditioned gating, calibrated confidence, and tests of recovery after the fault clears are needed to demonstrate graceful rather than accidental robustness.

Uncertainty should be propagated through the representation. Object-level covariance, occupancy probabilities, evidential mass, ensemble disagreement, or calibrated predictive distributions can express residual ambiguity. The choice of uncertainty method matters less than calibration under the relevant corruption and whether downstream planning uses the uncertainty. A numerically precise uncertainty estimate that is ignored by the planner does not provide graceful degradation. Table 13 summarizes representative multimodal architectures and their open reliability questions.

## 5. Adverse Weather, Contamination, and Interference

Adverse weather is not a single domain. Fog is dominated by suspended droplets and reduced contrast; rain introduces streaks, splashes, wet surfaces, and radar/lidar attenuation; snow creates particles, accumulation, and road-boundary loss; dust produces optical obscuration and scattering; and low sun produces glare. Performance also depends on intensity, droplet size distribution, wind, temperature, sensor heating, cleaning, target range, and target material. A statement that one modality “works in rain” is incomplete without these parameters.

The physical mechanisms also interact. Rain produces optical attenuation, lens droplets, road spray, specular reflections, LiDAR backscatter, and changes in radar clutter at the same time. Snow can create airborne returns, cover lane markings, block sensor windows, and alter the road surface used by localization. Fog density varies spatially and temporally, making a scene recorded at one visibility range a poor substitute for another. Consequently, a robustness claim should specify the environmental variables, their measurement method, and whether the sensor itself was exposed or the corruption was added digitally [17,18,19,28,36,37,38,39].

Public datasets such as Seeing Through Fog, CADC, RADIATE, Oxford RobotCar, and ACDC broaden environmental coverage [15,17,18,19,28]. Nevertheless, many weather datasets contain limited numbers of severe events, geographic regions, or rare objects. Controlled facilities enable repeatable intensity but may use short ranges and simplified targets. Simulation enables dense labels and parameter sweeps but depends on the fidelity of optical, electromagnetic, and particle models. Robust conclusions require triangulation across real road data, controlled experiments, and validated simulation.

Contamination is especially underrepresented. A few droplets, a smeared film, insect residue, mud, frost, or partial snow cover can create structured, spatially persistent corruption. Random pixel noise or uniform point dropout does not model these mechanisms. Sensor placement, airflow, heating, hydrophobic coatings, wipers, washer jets, and self-cleaning hardware are part of the sensing system and should be reported. Health monitoring should identify both complete obstruction and local regions where evidence is unreliable.

Cleaning and heating systems should be evaluated as part of the sensing architecture rather than treated as external maintenance. Their activation latency, coverage, energy use, fluid availability, thermal limits, and diagnostic feedback determine how long a modality remains degraded. A washer may remove loose dirt but fail on dried salt; heating may clear ice while increasing power demand or creating temporary optical distortion. The health monitor should verify restoration instead of assuming that an actuator command succeeded, and the vehicle should retain an ODD restriction until the recovered measurements satisfy defined acceptance criteria.

Environmental testing should include the transition into and out of a degraded state. Many datasets contain stable periods labeled as rain, fog, snow, or night, whereas a real vehicle encounters rapidly changing spray, sun exposure, tunnel entrances, condensation, and intermittent obstruction by other traffic. Transition sequences are particularly valuable for evaluating detection latency, hysteresis, and recovery. They can reveal whether temporal models continue to propagate obsolete evidence, whether confidence falls before accuracy, and whether the system restores full capability too quickly after the apparent condition has cleared.

Electromagnetic and cyber-physical interference add another dimension. Radar mutual interference can raise the noise floor or create false targets; GNSS can be jammed or spoofed; LiDAR can be affected by other emitters or deliberate optical injection; cameras can be dazzled; and communication-based sensing can be delayed or falsified. Security controls and perception robustness should therefore be co-engineered. An authenticated message may still be physically inaccurate, while a physically plausible sensor return may be maliciously induced.

Controlled environmental testing and naturalistic datasets provide different evidence. Chambers and spray rigs can sweep fog visibility, rain rate, droplet distribution, or contamination coverage while holding other factors constant, but they may not reproduce traffic-generated spray, wind, road heat, or heterogeneous targets. Naturalistic data capture realism but often lack precise severity labels and balanced repetitions. Strong evidence combines both: controlled tests establish a response curve and field data test external validity [18,19,28,36,37].

Corruption should be parameterized in physical terms whenever possible. Examples include the meteorological optical range for fog, rainfall or snowfall rate, percentage of aperture occlusion, angular extent of blocked LiDAR sectors, timing offset in milliseconds, radar interference-to-noise ratio, GNSS pseudorange bias, or IMU bias ramp. Performance plotted against severity reveals graceful degradation and operational thresholds more clearly than a binary clean-versus-corrupted comparison.

Common corruptions can also be correlated. Snow may attenuate LiDAR, obscure camera lenses, remove lane markings, and reduce tire friction simultaneously. A multimodal system trained with independent random dropout may therefore overestimate redundancy. Scenario-based evaluation should include joint failure distributions and recovery actions such as cleaning, heating, sensor reset, or route/ODD restriction. Table 14 links the resulting failure modes to diagnostic evidence and operational responses.

## 6. Sensor Health Monitoring and Graceful Degradation

Sensor health is a latent state that cannot be inferred reliably from a single statistic. It includes the hardware status, optical or electromagnetic path quality, calibration validity, timing integrity, environmental observability, and task relevance. The distinction between sensor health and task confidence is crucial. A camera can be healthy but unable to see through dense fog; a radar can be healthy but unable to resolve two adjacent pedestrians; and a LiDAR can have a blocked rear sector that is irrelevant for forward highway driving but critical for reversing.

These three outputs operate on different evidence. Sensor health can be estimated from internal diagnostics, signal statistics, environmental context, and cross-sensor consistency. Task confidence depends on the current object geometry, maneuver horizon, map context, and the capability of the remaining modalities. Residual observability is a system-level judgement about whether the vehicle can still establish the facts required for a bounded action. Combining them into one neural confidence score makes it difficult to determine why the system is uncertain and which fallback is justified [33,34,35,36,37,38,39,40,41,42,43].

A practical architecture should estimate quality at multiple levels. Signal-level indicators describe noise, saturation, return strength, frame completeness, temperature, packet loss, and timing. Geometric indicators test calibration, ego-motion, road plane, map consistency, and cross-sensor alignment. Perception-level indicators examine detection stability, innovation residuals, epistemic or aleatoric uncertainty, and disagreement among independent models. Operational indicators translate these estimates into the observable range, field-of-view coverage, object classes, and maneuver constraints.

Fault detection studies increasingly use confidence evaluation, fault injection, and simulation to expose how perception degradation propagates into vehicle behavior [40,41,42]. The evidence remains limited, however, because many experiments inject complete sensor loss rather than realistic partial degradation. A black camera frame is easy to detect; a more difficult case is gradual contrast loss, a single missing LiDAR sector, radar interference that creates plausible moving ghosts, or a 70 ms timestamp offset. Future benchmarks should contain severity-controlled, physically interpretable faults and should report both perception and system-level consequences.

Graceful degradation means preserving the maximum defensible function while reducing risk. It does not mean continuing normal operation with fewer sensors. The system may lower speed, increase headway, avoid lane changes, disable automated parking, restrict routes, request a transition, or reach a minimal-risk condition. The response should depend on residual observability, traffic context, ODD, and the expected time to recover. Safety standards emphasize systematic hazard analysis, functional safety, and intended-functionality limitations, but perception research must provide measurable evidence that supports these arguments [33,34,35,43].

The transition logic should include hysteresis and persistence to avoid oscillation between nominal and degraded modes. A single anomalous frame may not justify a braking response, whereas a slowly deteriorating camera can remain below a hard fault threshold for a long period. Multi-level thresholds can distinguish warning, restricted operation, and minimum-risk conditions, with different requirements for confirmation and recovery. Recovery should be treated as a controlled transition: the system may require several consistent observations, recalibration checks, and track reinitialization before restoring full maneuver capability.

**Figure 3:**
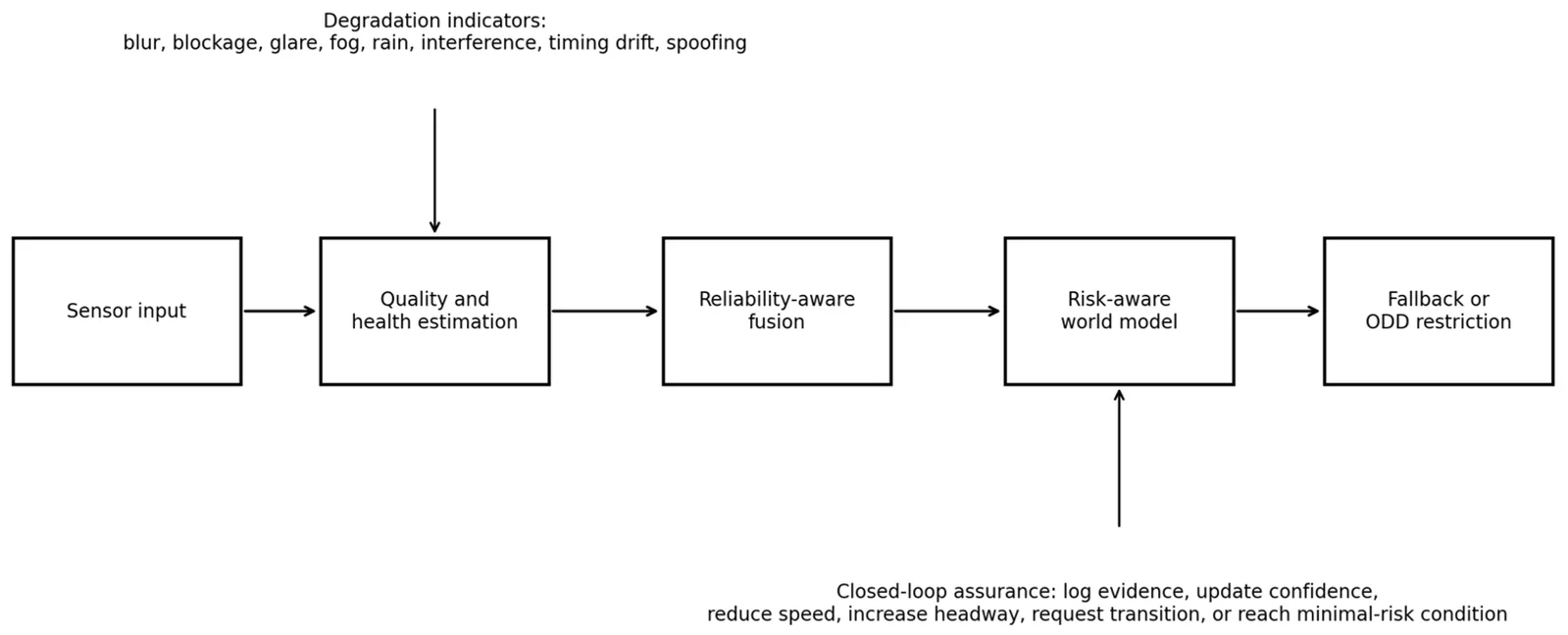
Closed-loop graceful degradation for safety-critical perception. The conceptual control loop synthesizes functional-safety, SOTIF, uncertainty, confidence-monitoring, and sensor-fault evidence [33,34,35,40,41,42,43].

A reliability-aware world model should carry uncertainty and provenance forward to planning. When evidence becomes inconsistent, the architecture should avoid averaging contradictory observations into a confident estimate. Instead, it should maintain competing hypotheses, expand covariance, lower object-existence probability only when justified, or conservatively preserve a potential obstacle. The correct policy depends on asymmetric risk: a false negative pedestrian is generally more severe than a temporary false positive that causes modest deceleration. Figure 3 illustrates the corresponding closed-loop graceful-degradation process.

### Proposed Reliability-Aware Architecture

The proposed architecture separates five functions. First, modality-specific monitors estimate raw signal validity and environmental quality. Second, calibration and timing monitors establish whether observations are geometrically and temporally comparable. Third, reliability-aware fusion conditions feature or hypothesis weights on health estimates and preserves uncertainty. Fourth, a risk-aware world model represents which regions, object classes, and motion states remain observable. Fifth, a supervisory function maps residual observability to ODD restrictions, transition requests, or minimum-risk maneuvers.

Evidence was mapped to each architectural function at three levels: direct experimental support, indirect component-level support, and synthesis-level recommendation. Health estimation has direct but limited evidence in confidence and fault-injection studies [40,41,42]. Fusion studies support multimodal association and robustness [62,63,64,65,66,67,68,69,77,78,79], but most do not directly validate health-conditioned reweighting. ODD restriction and minimum-risk behavior are primarily normative or synthesis-level extensions grounded in SAE/ISO/UL safety concepts [1,33,34,35,43], with only sparse system-level testing [41,42].

Implementation of this architecture benefits from a contract between perception and planning. Perception should expose not only objects and lanes but also coverage gaps, unsupported regions, observation age, and the health state of the contributing sensors. Planning should define which evidence is mandatory for each maneuver and how speed, headway, lane-change availability, and route selection change when the contract is not satisfied. This avoids a generic “degraded perception” flag and creates a traceable link between sensor diagnostics and vehicle behavior.

Validation should include false alarms as well as missed degradation. An over-sensitive monitor can repeatedly restrict the ODD or trigger unnecessary minimum-risk maneuvers, creating availability and traffic-safety problems. Conversely, a monitor that is tuned only for severe faults may miss gradual contamination or plausible corrupted features. Performance should therefore be reported using detection delay, sensitivity, specificity, false-warning rate per operating hour, severity at detection, and the downstream risk reduction achieved by the selected response [40,41,42,43].

The health architecture should also be robust to disagreement between its own monitors. An internal diagnostic may report normal hardware operation while scene statistics indicate loss of observability, or a cross-modal residual may increase because the environment affects both sensors differently rather than because either sensor is faulty. A hierarchical decision process can retain these competing hypotheses, request additional evidence over a short temporal window, and select a conservative response when the cause cannot be isolated. This is preferable to forcing every anomaly into a single fault class and allows functional insufficiency to be handled even when the transducer remains electrically healthy [33,34,35,36,37,38,39,40,41,42,43].

Sensor-health estimation and task confidence answer different questions. Health estimation asks whether a measurement channel behaves as expected given the hardware status and environmental context. Task confidence asks whether the current world-model output is sufficiently supported for a maneuver. A camera can be healthy in dense fog while providing insufficient long-range evidence; conversely, a partially obstructed camera may remain adequate for a low-speed reverse maneuver. The decision layer must therefore map local sensor observability to task-specific requirements.

Fault detection requires explicit false-alarm analysis. Aggressive thresholds may trigger frequent ODD restrictions during legitimate sparse scenes, while permissive thresholds delay intervention. Papers should report the detection probability, false-alarm rate, time to detection, severity at detection, recovery time, and the resulting perception and vehicle-level performance. These metrics should be evaluated for abrupt faults and gradual degradation [40,41,42]. Table 15 summarizes representative health indicators, hypotheses, cross-checks, and responses.

The traceability mapping prevents the conceptual architecture from being presented as experimentally validated. A planner-level example can illustrate how health estimates might justify speed reduction, but the corpus directly supports only parts of that chain. Vehicle-level validation of thresholds, ODD restrictions, false alarms, recovery, and minimum-risk outcomes remains a research requirement.

## 7. Datasets, Benchmarks, and Evaluation Metrics

Benchmark selection shapes conclusions. A method trained and tested on one city with a fixed sensor suite may learn local priors, calibration patterns, and object distributions. Cross-dataset evaluation is therefore more informative for robustness than small within-dataset gains. Weather and corruption results should report severity, range, object class, sensor configuration, and whether calibration or retraining was adapted to the target domain. Table 16 compares representative datasets and their principal benchmark limitations.

Benchmark composition should be analyzed at the level of scenarios and sensor states. A large number of frames does not guarantee diversity if most are recorded in similar daylight, road, and traffic conditions. Sequence-level splitting is necessary to avoid near-duplicate leakage, and geographic separation is important when the map structure, architecture, lane markings, or weather is correlated with the training set. For degradation research, metadata should identify the visibility, precipitation rate, surface spray, sun angle, contamination state, sensor temperature, packet loss, and calibration status rather than relying on broad labels such as “rain” or “night.”

Mean average precision and intersection-over-union remain useful task metrics, but safety evaluation needs additional measures: detection probability as a function of range and weather; false-negative and false-positive risk by object class; calibration error; expected calibration error or negative log likelihood; track continuity; localization integrity risk; time-to-detection; stale-data rate; end-to-end latency; minimum observable stopping distance; and system behavior after fault onset. A complete evaluation should include both nominal performance and degradation curves rather than a single average value.

Robustness results should be presented as performance–severity curves with uncertainty intervals, not only as clean-versus-corrupted averages. The evaluation should distinguish difficult in-distribution scenes, distribution shift, and actual sensor impairment. Missing-modality tests should include partial and intermittent loss, and recovery should be measured after the input returns. Where possible, benchmark metrics should be connected to a scenario requirement, such as detection probability within the stopping horizon, localization protection level relative to lane width, or false-positive persistence that could provoke unnecessary braking [9,10,11,12,13,14,15,16,17,18,19,20,21,22,23,24,25,26,27,28,29,30,36,37,38,39,40,41,42,43].

Benchmark protocols should define how models are selected and tuned. When a corruption benchmark is used repeatedly for architecture development, the nominal test set can become an implicit training resource even without direct gradient updates. Hidden evaluation conditions, fixed public severity definitions, and a separate development set can reduce this form of overfitting. Reporting should also include whether calibration, thresholds, or normalization statistics were recomputed after corruption. A method that adapts using information from the complete test sequence is not directly comparable with a causal online system that must react without future observations.

Benchmark comparison also requires range and class stratification. A single aggregate mAP can conceal poor detection of distant pedestrians or small obstacles, while average localization error can conceal hazardous tail events. Results should be reported by distance, visibility, object size, occlusion, weather severity, road type, and sensor-health state. For temporal systems, metrics should include track fragmentation, time-to-detection, and persistence of false objects.

Dataset design should expose the sensor state. In addition to object annotations, future corpora should label lens contamination, LiDAR sector blockage, radar interference, timestamp validity, calibration state, cleaning events, and estimator integrity. Without these labels, a health-monitoring method cannot be evaluated independently from task accuracy, and false alarms cannot be attributed to context or true hardware degradation. Table 17 lists the recommended reliability-centered evaluation metrics.

## 8. Discussion

### 8.1. Why No Single Modality Is Sufficient

The synthesis supports a physically grounded conclusion: every sensing modality has conditions under which its evidence becomes weak, ambiguous, delayed, or misleading. Camera-only systems can achieve a strong nominal perception but remain coupled to optical visibility and appearance generalization. LiDAR-centric systems obtain accurate geometry but face point sparsity, scattering, contamination, and cost. Radar-centric systems preserve velocity and weather resilience but face angular ambiguity and multipath. The decision to deploy one or more modalities is therefore not solely an accuracy or cost question; it is a claim about the ODD, acceptable residual risk, maintenance, and fallback capability.

The benefit of heterogeneity is conditional on the operational design domain. A low-speed parking system may obtain sufficient coverage from cameras and ultrasonic sensors, while high-speed motorway automation requires longer-range geometry and velocity evidence. Dense urban driving places greater value on semantics, occlusion reasoning, and vulnerable-road-user detection, whereas poor-visibility operation increases the importance of radar and active illumination. Sensor selection should therefore begin with scenario-level information requirements and failure tolerance rather than a universal modality ranking [6,7,8,18,19].

Modality sufficiency should also be evaluated over the complete maneuver sequence rather than at a single perception instant. A sensor combination that supports lane keeping may not provide the side and rear observability needed to initiate, execute, and abort a lane change. Similarly, a configuration that detects a stationary obstacle at the required range may still lack the semantic or velocity evidence needed to predict a vulnerable road user. Scenario decomposition should therefore identify the information required before, during, and after each maneuver, together with the time available to recognize degradation and transition to a safer state. This perspective turns sensor selection from a component comparison into a temporal coverage problem and makes clear why the same architecture can be adequate for one ODD function but insufficient for another.

Diverse sensing can reduce risk only when the architecture recognizes which failures are independent. Rain simultaneously affects road appearance, camera contrast, LiDAR scattering, and radar clutter, although by different mechanisms. A power or network fault can disable several sensors at once. Sensor cleaning may restore optics but not calibration. Redundancy analysis should include common-cause failures, shared processing hardware, shared clocks, shared mounts, and shared learned representations.

### 8.2. Accuracy Is Not Reliability

Benchmark accuracy measures average task performance under a specified data distribution. Reliability requires predictable behavior across time, uncertainty, faults, and distribution shift. A model can improve mean average precision while becoming more overconfident under corruption. Conversely, a slightly less accurate model with calibrated uncertainty and stable degradation may be preferable for safety. Papers should therefore report performance–severity curves, confidence calibration, failure detection latency, and the operational consequences of errors.

Reliability also includes the calibration of uncertainty. A model can lose accuracy gradually while remaining highly confident, which is more dangerous than an explicit low-confidence output. Calibration should be evaluated across environmental severity, object range, class, occlusion, and sensor state because a single expected calibration error can hide local overconfidence. The downstream system must also know whether uncertainty reflects ambiguous scene content, limited range, missing coverage, or suspected sensor corruption, since these causes justify different operational responses [38,39,40,41,42,43].

Reliability is furthermore affected by how a model behaves outside the conditions represented during training. A network may retain acceptable average accuracy while its errors become concentrated in rare combinations of range, weather, object class, and occlusion. Aggregate robustness metrics can therefore obscure safety-critical pockets of failure. Evaluation should stratify results by scenario factors and examine whether confidence deteriorates before task performance becomes unacceptable. When uncertainty is not predictive of error, the system needs independent monitors or conservative operating limits rather than a lower decision threshold. This is especially important for deep fusion models, where correlated feature errors can produce internally consistent but incorrect outputs that are difficult to identify from the final detection score alone [38,39,40,41,42,43].

Missing-modality robustness is often demonstrated by dropping an entire stream during training or testing. This is useful but insufficient. Real sensor degradation is frequently partial and structured: one camera quadrant is blocked, several LiDAR rings are missing, radar interference appears intermittently, or timestamps drift gradually. Models trained only with random modality dropout may fail to identify these conditions because the corrupted stream remains plausible. Health-aware gating should use explicit diagnostics rather than infer all validity implicitly from task loss.

### 8.3. Deployment and Computational Constraints

High-resolution sensing and fusion increase bandwidth, memory, power, heat, and latency. Raw radar and dense LiDAR preserve information but can overwhelm embedded platforms. Multi-camera BEV transformers require substantial computation and can introduce temporal delay. The safety-relevant quantity is not network inference time alone, but sensor exposure or scan time, transfer, preprocessing, synchronization, inference, tracking, and publication to planning. Energy and thermal throttling can also change performance during long operation.

Compute scheduling can become a common-cause failure. Camera, LiDAR, and radar branches may be physically independent but still share a GPU, memory bus, network switch, or thermal envelope. Under overload, delayed outputs from several modalities can appear mutually consistent while all are too old for the current maneuver. Safety evaluation should therefore include worst-case execution time, queue growth, deadline misses, resource contention, and the behavior of the fusion and planner when one branch is intentionally deprioritized.

Resource isolation and degraded-mode computation should be designed together. A fallback perception path is not credible if it requires the same saturated accelerator, memory allocation, or software service that caused the primary pipeline to miss deadlines. Systems can reserve compute for health monitoring and minimum-risk perception, use simpler modality-specific models, reduce spatial resolution or frame rate in a controlled manner, and prioritize the region needed for the immediate fallback maneuver. These choices should be validated under thermal throttling and communication congestion, because graceful degradation may otherwise exist only in a nominal laboratory configuration. The relevant performance measure is not peak throughput but whether the minimum required evidence is produced before the planning deadline under the defined worst-case resource state.

Cost reduction should be evaluated against lifecycle assurance. Additional sensors increase the purchase price, cleaning, calibration, and service complexity, but they may also provide observability needed for fallback. Conversely, adding a sensor without independent processing or health monitoring can create only nominal redundancy. Architecture comparison should therefore include the bill of materials, power, failure independence, maintainability, and the ODD that remains available after a single fault.

Deployment comparison should include the bandwidth and data-retention strategy. Raw sensing maximizes diagnostic and reprocessing capability but increases compute, storage, privacy, and cybersecurity exposure. Aggressive preprocessing reduces bandwidth but may remove evidence needed to diagnose a ghost target or contamination event. A defensible design documents where information becomes irreversible and which diagnostic summaries remain available after that point.

Energy-aware redundancy is an emerging requirement. Heating, cleaning, high-resolution cameras, dense LiDAR, raw radar processing, and multi-encoder fusion can create significant thermal load. A system should demonstrate that its safety-critical sensor configuration remains available under sustained operation, hot-weather derating, a low battery state, and degraded cooling, rather than assuming nominal compute and power indefinitely. Table 18 summarizes the corresponding deployment and safety-case evidence.

### 8.4. Redundancy Diversity and Common-Cause Failure

Redundancy should be evaluated by independence of evidence, not by sensor count. Two cameras behind the same windshield may share glare, condensation, mud, power, compute, and timebase failures. Two LiDARs can share fog attenuation or cleaning-system loss. Camera–LiDAR–radar diversity reduces some common causes because the modalities use different physical principles, yet they may still share mounting vibration, network congestion, thermal constraints, or corrupted calibration. A failure-dependency matrix should therefore accompany the sensor architecture.

Diverse redundancy also creates disagreement. When radar preserves a moving-object hypothesis that camera and LiDAR do not confirm, the system must decide whether radar is detecting through obscuration or producing multipath. Majority voting is not sufficient because two degraded modalities can outvote a valid third. Reliability-aware reasoning should use environmental context, sensor-health indicators, historical consistency, and asymmetric safety costs.

A structured disagreement policy can improve both safety and diagnosability. The system can classify conflicts by object type, range, persistence, environmental context, and expected modality capability. A radar-only moving target in dense spray may be retained with conservative geometry, whereas a transient radar-only stationary target near a reflecting barrier may receive lower confidence. The policy should be tested against asymmetric errors because discarding a valid vulnerable road user and preserving a harmless ghost do not have the same consequence.

Common-cause analysis should be performed systematically rather than inferred from the use of different sensor names. A dependency model can include a shared aperture location, contamination exposure, mounting structure, time synchronization, power supply, communication links, compute resources, calibration procedures, map sources, and training data. Fault-tree or dependency-matrix analysis can then identify combinations that defeat nominal redundancy and reveal where architectural separation is needed. The analysis should also include maintenance-induced causes, such as a misaligned replacement sensor or a cleaning system that services several apertures. Diversity at the transducer level is valuable, but independence must be demonstrated across the complete acquisition and processing chain.

### 8.5. Standards and Safety-Case Integration

ISO 26,262 addresses malfunctioning behavior of electrical and electronic systems, while ISO 21,448 addresses hazards arising from functional insufficiencies and foreseeable misuse [33,34]. UL 4600 provides a safety-case-oriented framework for autonomous products [35]. Sensor research can support these processes by defining performance envelopes, diagnostic coverage, fault models, uncertainty bounds, and scenario-based evidence. However, a dataset leaderboard is not a safety case because it does not establish the completeness of hazards, independence of redundancy, or operational response.

A traceable assurance argument can connect sensor specifications to scenario requirements. For example, a highway ODD may require reliable obstacle evidence beyond the stopping-distance horizon under specified rain intensity and road spray. Test evidence should show the detection probability, false alarms, latency, health-monitor performance, and fallback behavior when one modality falls below the requirement. Similar claims can be formulated for lane boundaries, vulnerable road users, localization integrity, and low-speed near-field obstacles.

The assurance case should also address change management. Replacing a sensor, updating preprocessing, retraining a fusion network, or altering calibration software can invalidate earlier evidence even when interface dimensions remain unchanged. Configuration control should link each safety claim to hardware revision, software version, dataset, test scenario, and acceptance threshold. Post-deployment monitoring can then detect whether field conditions fall outside the validated envelope and provide evidence for targeted updates rather than relying only on periodic aggregate performance [33,34,35,43].

A strong safety argument distinguishes evidence for the sensor, the perception algorithm, the health monitor, and the operational response. For each claim, the argument should state the assumed environment, target characteristics, range, timing budget, diagnostic coverage, and residual uncertainty. It should then show that a detected limitation leads to a bounded restriction before the corresponding hazard becomes uncontrollable. This claim-evidence structure prevents a general benchmark result from being used to support an unrelated ODD condition and clarifies which tests must be repeated after a hardware or software change. It also creates a practical interface between research metrics and safety engineering, because each experimental result has an explicit role rather than serving as undifferentiated evidence of “robustness” [33,34,35,43].

## 9. Research Agenda

The most important shift is from clean-dataset accuracy toward evidence about degradation. New models should be evaluated not only by how well they fuse sensors when all inputs are valid but also by whether they detect invalid evidence, maintain calibrated uncertainty, and trigger an appropriate operational response. This requires datasets and simulators to expose sensor-state labels, not merely object labels. Table 19 organizes the resulting research priorities.

A second priority is to connect perception research with vehicle-level decisions. A health monitor that identifies camera blur is useful only if the system can determine whether remaining sensors provide enough range and semantics for the current maneuver. Joint evaluation across sensing, world modeling, planning constraints, and fallback will make research claims more relevant to SOTIF and safety assurance.

Finally, the community needs clearer reporting. Sensor model and placement, calibration, synchronization, weather severity, preprocessing, runtime platform, train/test split, uncertainty method, and failure-injection mechanism should be mandatory. Without these details, reproducibility and cross-paper comparison remain weak even when source code is available.

A third priority is cross-hardware validation. Models trained on one camera response curve, LiDAR scan pattern, or radar processing chain may not transfer to another. Public benchmarks should therefore include multiple hardware generations or provide standardized intermediate representations together with raw data. Domain adaptation should be evaluated separately from sensor-health detection so that a distribution shift is not confused with a physical fault.

A fourth priority is benchmark governance. Corruption severity, evaluation code, hidden test sets, metadata quality, and version history should be controlled to prevent unintentional leakage and incomparable claims. Safety-relevant leaderboards should include calibration, latency, robustness, and missing-modality results rather than ranking a single nominal metric.

Finally, research should connect uncertainty to action. The practical output of perception is not merely an object list but a bounded statement about what is known, unknown, and sufficiently observable for the current maneuver. Demonstrating that uncertainty changes speed, headway, lane-change availability, route choice, or minimum-risk behavior would make perception studies more relevant to vehicle-level assurance.

A fifth priority is the creation of open sensor-health benchmarks. Such benchmarks should combine physically measured degradation with validated simulation, include gradual and sector-specific faults, and provide synchronized labels for sensor state, environmental state, perception output, and vehicle response. They should support evaluation of detection, isolation, adaptation, and recovery rather than only final task accuracy. Shared protocols would make it possible to compare health monitors independently of a particular fusion network and to identify which failure mechanisms remain poorly covered.

A sixth priority is lifecycle evidence. Sensor performance changes through aging, cleaning cycles, repairs, mounting tolerances, software updates, and seasonal operation. Longitudinal fleet studies could reveal failure frequencies, warning precursors, and false-alarm costs that short benchmark experiments cannot estimate. Privacy-preserving field logs containing health summaries, trigger events, and anonymized scenario descriptors would help connect laboratory robustness to real operational reliability.

A seventh priority is human- and operations-aware fallback. Minimum-risk maneuvers occur in traffic and can create secondary hazards when they are abrupt, frequent, or poorly communicated. Research should evaluate how sensor degradation influences transition timing, external signaling, remote assistance, passenger information, and the selection of a stopping location. The objective is not to transfer the dynamic driving task to an unprepared human, but to ensure that the automated response remains predictable and compatible with surrounding road users. Integrating these operational factors would broaden graceful degradation from a perception property into a complete vehicle capability.

## 10. Practical Design and Reporting Recommendations

For system designers, the review supports a four-part rule. First, specify the sensing requirement from the driving scenario and stopping or maneuver horizon rather than selecting sensors from generic range tables. Second, represent each sensor’s health, observation age, calibration state, and uncertainty independently from the perception output. Third, preserve provenance through the fused world model so that fallback can reason about residual evidence. Fourth, connect health and uncertainty thresholds to explicit ODD restrictions and minimum-risk behavior.

For researchers, every study should state the sensor model and placement, preprocessing, synchronization, calibration, dataset split, environmental conditions, fault mechanism and severity, runtime platform, and uncertainty method. Results should include unimodal baselines, sensor-ablation and partial-degradation tests, performance-severity curves, and false-alarm analysis. Claims should distinguish benchmark robustness from demonstrated vehicle-level safety.

For reviewers and editors, claims should be matched to the level of evidence. A study using one public dataset can support a benchmark claim, but not universal weather robustness. A controlled fog chamber can establish a physical performance relationship, but not complete ODD coverage. A fault-injection simulator can compare adaptation strategies, but its safety meaning depends on the realism and validation of the injected fault. Explicitly labeling these evidence levels would reduce overstatement and make systematic comparison more transparent.

For dataset developers, the highest-value addition is often not another nominal sequence but better metadata and controlled variation. Recording calibration files, sensor timestamps, dropped packets, cleaning events, environmental measurements, hardware revisions, and known sensor anomalies can transform a perception dataset into a reliability resource. Benchmark governance should preserve hidden test integrity while publishing enough scenario and severity information to support scientific interpretation.

For authors preparing a systematic or experimental *Sensors* submission, the reporting checklist should be used during study design rather than only at manuscript completion. Predefining the sensor-state variables, severity levels, comparison baselines, exclusion rules, and runtime measurements reduces selective reporting and makes negative robustness results publishable. Tables should separate hardware properties from algorithmic settings and should identify which conclusions are directly measured, which are derived from ablation, and which are proposed design implications. Supplementary Material is an appropriate location for complete calibration files, fault-injection parameters, scenario lists, and per-severity results, but the main article should still contain enough information to permit an understanding of the validity and scope of every principal claim. Table 20 provides the minimum reporting checklist derived from these requirements.

## 11. Limitations

This review synthesizes heterogeneous engineering evidence and therefore does not calculate a single pooled effect. Primary studies use different datasets, hardware, metrics, operating conditions, and corruption severities. Although the final PRISMA counts, 65-study matrix, and row-level methodological appraisal are reconciled, the review remains limited by single-reviewer two-pass screening, extraction, and appraisal. The earlier claim of independent duplicate screening was removed; no inter-reviewer kappa can be calculated without an independent decision file. The review was retrospectively registered in PROSPERO on 30 July 2026 (CRD420261465869), after pilot work, formal searching, and screening had already been undertaken. This registration chronology increases the possibility of post hoc methodological decisions and should be considered when comparing the published review with the registered record. These limitations increase the risk of selection and coding error and should be considered when interpreting the synthesis.

The review also focuses on road vehicles. Findings from robotics, aviation, maritime autonomy, and rail sensing were excluded unless directly validated for road-driving perception. Hardware evolves rapidly, particularly imaging radar, solid-state LiDAR, and event cameras, so cost and performance comparisons can become outdated. For this reason, the review emphasizes physical failure mechanisms, evidence requirements, and architectural principles that are more stable than specific product specifications.

The qualitative synthesis is additionally limited by reporting bias. Studies are more likely to publish successful fusion methods than failed approaches, and proprietary sensor preprocessing can prevent reconstruction of the measurement chain. The review did not infer missing hardware or corruption parameters from product documentation. As a result, some comparisons remain at the architectural rather than numerical level. These limitations reinforce the need for standardized reporting and do not justify ranking modalities by isolated headline metrics.

Search limitations remain. English-language and peer-reviewed publication requirements may omit industrial or regional evidence, and terminology differences can place reliability studies outside autonomous-driving indexes. Citation chaining reduces but does not eliminate this risk. The final database search was completed on 29 July 2026; because revision occurred 17 days later, no substantial update interval had elapsed, but the search should be rerun if editorial processing extends materially beyond this date.

## 12. Conclusions

Across 65 included primary studies, no sensing modality demonstrated universal reliability across operational design domains. Camera, LiDAR, radar, thermal/event imaging, and inertial/localization sources provide complementary but condition-dependent evidence. Their value depends on hardware, environment, range, calibration, timing, processing, and the downstream task; the review therefore does not rank modalities universally.

Direct evidence is concentrated at the component and benchmark levels. Public datasets and fusion architectures demonstrate the task performance within specified configurations, while realistic contamination, gradual degradation, interference, sensor-health labels, calibrated uncertainty, and vehicle-level fallback remain comparatively sparse. Nominal accuracy must therefore not be interpreted as evidence of operational reliability.

The strongest evidence-supported conclusion is that sensor health, task confidence, and residual observability are distinct quantities. A healthy sensor may be uninformative in a specific environment, while a degraded sensor may still support a bounded low-speed maneuver. Reliable fusion must preserve this distinction and expose uncertainty, timing, coverage, and provenance to the decision layer.

The reliability-aware architecture is consequently presented as a synthesis-level design framework, not as a system validated by all included studies. Health estimation has limited direct support [40,41,42]; multimodal fusion provides indirect support for adaptation [62,63,64,65,66,67,68,69,77,78,79]; and ODD restriction and minimum-risk behavior are grounded mainly in safety standards and conceptual assurance arguments [1,33,34,35,43].

Future work should prioritize physically parameterized degradation, cross-hardware and cross-dataset validation, uncertainty calibration, false-alarm and recovery metrics, end-to-end latency and energy reporting, and explicit links from sensor state to maneuver availability. These additions are necessary before a complete health-aware degradation loop can be claimed as operationally validated.

In conclusion, dependable automated driving requires not only accurate perception but also defensible knowledge of when sensory evidence is insufficient. The principal contribution of this review is a traceable separation between what the 65-study corpus directly demonstrates and what is proposed as an architecture for future validation.

## Figures and Tables

**Table 1:** Eligibility criteria applied to titles, abstracts, and full texts. Criteria were adapted from PRISMA reporting principles and the autonomous-driving scope defined by SAE J3016 [1,45,46].

Criterion	Included	Excluded
Publication type	Peer-reviewed journal articles and full conference papers; standards and authoritative reports used for contextual discussion.	Abstract-only records, posters without full methods, patents, marketing material, news reports.
Domain	Automated road vehicles, ADAS perception directly transferable to automated driving, vehicle localization, cooperative perception.	Unmanned aerial vehicles, marine robots, indoor robots unless the method is validated on road-driving data.
Sensor scope	RGB/stereo/thermal/event camera, LiDAR, radar, ultrasonic, GNSS/RTK, IMU/INS, wheel odometry, V2X sensing, multisensor fusion.	Studies without a sensing, localization, calibration, failure, fusion contribution.
Outcomes	Detection, segmentation, tracking, free-space/occupancy, localization, calibration, robustness, uncertainty, health monitoring, failure mitigation, latency.	Pure planning/control studies with no sensor or perception evaluation.
Language and period	English; January 2015–July 2026, plus foundational earlier works required to explain datasets, localization, weather effects, or standards.	Non-English full text when no reliable translation is available.
Evidence quality	Methods and evaluation sufficiently described to extract sensor configuration, data source, and at least one performance or safety-relevant result.	Opinion pieces, unvalidated conceptual claims.

**Table 2:** Conceptual search blocks translated to the syntax of each database interface. The structure follows PRISMA-S requirements for reproducible literature searches [46]. The asterisk (*) is the database truncation wildcard used to retrieve word variants.

Block	Search Terms
Core domain	(“autonomous vehicle*” OR “automated driving” OR “self-driving” OR “driverless vehicle*”)
Sensor modality	(sensor* OR camera* OR vision OR lidar OR LiDAR OR radar OR ultrasonic OR GNSS OR GPS OR IMU OR inertial OR thermal OR “event camera”)
Fusion/perception	(“sensor fusion” OR multimodal OR “multi-sensor” OR perception OR localization OR mapping OR SLAM OR calibration OR synchronization)
Reliability/safety	(failure OR fault OR degradation OR robustness OR uncertainty OR contamination OR weather OR fog OR rain OR snow OR spoofing OR interference OR “health monitoring” OR redundancy OR “graceful degradation”)
Combined query	Core domain AND Sensor modality AND (Fusion/perception OR Reliability/safety).

**Table 3:** Final executed database searches, platform fields, filters, execution dates, and returned record counts. Queries are reproduced as run on 29 July 2026 in accordance with PRISMA-S [46]. The asterisk (*) is the database truncation wildcard used to retrieve word variants.

Database/Platform	Executed Fields, Filters, Date, and Count	Exact Executed Query/Search Route
Scopus	TITLE-ABS-KEY; English; article or conference paper; 29 July 2026; *n* = 712	TITLE-ABS-KEY((“autonomous vehicle*” OR “automated driving” OR “self-driving car*”) AND (camera* OR lidar OR radar OR ultrasonic OR thermal OR “event camera*” OR GNSS OR IMU OR “inertial measurement”) AND (perception OR localization OR calibration OR synchronization OR fusion OR degradation OR fault* OR robustness OR uncertainty))
Web of Science Core Collection	TS field; English; article or proceedings paper; 29 July 2026; *n* = 604	TS = ((“autonomous vehicle*” OR “automated driving” OR “self-driving car*”) AND (camera* OR lidar OR radar OR ultrasonic OR thermal OR “event camera*” OR GNSS OR IMU) AND (perception OR localization OR calibration OR synchronization OR fusion OR degradation OR fault* OR robustness OR uncertainty))
IEEE Xplore	All Metadata; journals and conferences; English; 29 July 2026; *n* = 458	((“All Metadata”:”autonomous vehicle” OR “All Metadata”:”automated driving”) AND (“All Metadata”:sensor OR camera OR lidar OR radar OR GNSS OR IMU) AND (fusion OR localization OR calibration OR fault OR degradation OR robustness))
ScienceDirect	Title/abstract/author keywords; research articles and conference papers; English; 29 July 2026; *n* = 369	(“autonomous vehicle” OR “automated driving”) AND (sensor OR camera OR lidar OR radar OR GNSS OR IMU) AND (perception OR localization OR fusion OR calibration OR degradation OR fault OR robustness)
Supplementary checking	Backward/forward citation checking; *n* = 0 additional primary-study records	Coverage checking and contextual-source identification; not added to the database identification total.
Total	Database records *n* = 2143; registers/other methods *n* = 0; overall *n* = 2143	Identification total before deduplication and pre-screening removals.

**Table 4:** Record-management and deduplication workflow. The procedure operationalizes PRISMA-S requirements for transparent record handling [46].

Stage	Decision Rule and Final Count	Audit Information Retained
Import	Combine four database exports; total *n* = 2143.	Database, date, platform, format, and original identifier.
Duplicate removal	Normalized DOI; otherwise, normalized title, year, and first author; *n* = 612 removed.	Duplicate pair, rule, retained record, and removed record.
Automation removal	Predefined language, year, and document-type rules; *n* = 86 flagged and removed only after manual verification.	Source identifier, rule triggered, bibliographic fields checked, manual confirmation, and final decision.
Other pre-screening removal	Non-article and irrecoverably incomplete bibliographic records; *n* = 95 removed.	Reason code and source identifier.
Title/abstract screening	All remaining records screened; *n* = 1350.	Decision, reason code, and repeat-check decision.
Full-text assessment	Reports sought *n* = 294; not retrieved *n* = 21; assessed *n* = 273; excluded *n* = 208; included *n* = 65.	Full-text decision and one mutually exclusive exclusion reason.

**Table 5:** Engineering-oriented methodological quality checklist used for sensitivity interpretation. The items reflect reproducibility, functional-safety, SOTIF, and uncertainty-reporting needs [6,7,33,34,43].

Item	Operational Interpretation	Score
Sensor configuration	Model, placement, field of view, resolution, rate, and range reported.	0/0.5/1
Calibration/synchronization	Extrinsic/intrinsic calibration and timestamp handling described.	0/0.5/1
Dataset provenance	Collection source, split, licensing, and geography stated.	0/0.5/1
Split integrity	Training, validation, and test separation prevents leakage.	0/0.5/1
Baseline adequacy	Relevant unimodal or fusion baseline included.	0/0.5/1
Metric validity	Task metric and operating threshold appropriate.	0/0.5/1
Corruption severity	Weather, blockage, noise, drift, or failure level quantified.	0/0.5/1
Runtime evidence	Latency, throughput, memory, or energy discussed.	0/0.5/1
Uncertainty	Variability, confidence calibration, or error bounds reported.	0/0.5/1
Reproducibility	Code/data available or implementation sufficiently specified.	0/0.5/1

**Table 6:** Quantitative profile of the 65 included primary studies by study type, sensor modality, dataset/resource class, evaluation environment, degradation category, and evidence level. Sensor and degradation categories are non-exclusive where indicated. The merged table also reports the verified domain-level methodological quality appraisal of the 65 included primary studies. Score distributions are reported in the order 1/0.5/0; corruption severity additionally reports N/A. Row-level judgments and calculation details are provided in Supplementary File S1.

**Coding Dimension**	**Category**	***n***	**Interpretation**
Study type (exclusive)	Dataset/benchmark resource	15	Public or documented sensing datasets and benchmark resources.
Study type (exclusive)	Perception/fusion algorithm	36	Detection, segmentation, BEV, representation, or multimodal fusion.
Study type (exclusive)	Localization/odometry	6	Radar, visual-inertial, or LiDAR-inertial estimation.
Study type (exclusive)	Degradation/fault evaluation	7	Weather/corruption benchmarking, confidence monitoring, or fault injection.
Study type (exclusive)	Simulation/data generation	1	LiDAR simulation used as a primary methodological contribution.
Sensor modality (non-exclusive)	Camera/visible imaging	45	Dominant in datasets, BEV, and fusion studies.
Sensor modality (non-exclusive)	LiDAR	35	Common in 3D detection, multimodal fusion, and localization.
Sensor modality (non-exclusive)	Radar	18	Fewer studies and heterogeneous representations.
Sensor modality (non-exclusive)	GNSS/IMU/inertial	10	Primarily dataset localization and state estimation.
Sensor modality (non-exclusive)	Thermal/gated/event	4	Specialized low-light, adverse-weather, or high-rate sensing.
Sensor modality (non-exclusive)	Ultrasonic	0	No eligible primary study; an explicit evidence gap.
Dataset/resource class (exclusive, *n* = 15)	General multimodal road datasets	6	KITTI-, nuScenes-, Waymo-, Argoverse-, and PandaSet-type resources.
Dataset/resource class (exclusive, *n* = 15)	Adverse/seasonal datasets	3	Long-term, winter, or adverse-weather resources.
Dataset/resource class (exclusive, *n* = 15)	Radar-specific datasets	2	Raw or processed automotive-radar resources.
Dataset/resource class (exclusive, *n* = 15)	Visual/semantic datasets	3	Large-scale visual or LiDAR semantic annotation resources.
Dataset/resource class (exclusive, *n* = 15)	Event-camera dataset	1	Driving event/frame/LiDAR resource.
Evaluation environment (exclusive)	Public real-world/recorded-road data	48	Largest evidence stratum; hardware and geography remain dataset-specific.
Evaluation environment (exclusive)	Private/limited road or laboratory data	8	Restricted scale or hardware-specific evidence.
Evaluation environment (exclusive)	Simulation/synthetic only	6	External validity depends on the fault or rendering model.
Evaluation environment (exclusive)	Mixed real and synthetic/controlled	3	Supports triangulation but uses nonuniform protocols.
Degradation category (non-exclusive)	Weather/visibility/optical corruption	11	Most developed degradation category.
Degradation category (non-exclusive)	Loss/blockage/packet or modality dropout	5	Often abrupt rather than gradual or sector-specific.
Degradation category (non-exclusive)	Calibration or timing drift	6	Usually discussed; less often evaluated as a progressive fault.
Degradation category (non-exclusive)	Radar interference/multipath	4	Sparse and hardware-dependent evidence.
Degradation category (non-exclusive)	GNSS degradation/spoofing	3	Limited direct vehicle-level validation.
Degradation category (non-exclusive)	No explicit degradation experiment	42	Nominal benchmark evidence should not be read as reliability evidence.
Evidence level (exclusive)	Level 1: component/benchmark	48	Direct task-level evidence within a defined evaluation setting.
Evidence level (exclusive)	Level 2: controlled degradation/diagnostic	12	Direct degradation or diagnostic evidence without full ODD response.
Evidence level (exclusive)	Level 3: operational/system response	5	Limited system-level evidence; no broad field validation of the proposed architecture.
**Quality Domain**	**Score Distribution, *n* (1/0.5/0; N/A Where Applicable)**	**Applicable *n***	**Interpretation**
Sensor configuration	31/33/1	65	31 studies were complete, 33 partial, and 1 unreported; many algorithm papers relied on dataset-level hardware descriptions.
Calibration/synchronization	32/31/2	65	32 studies were complete, 31 partial, and 2 unreported; partial scores commonly reflected inherited dataset calibration.
Dataset provenance	59/6/0	65	59 studies were complete and 6 partial; provenance was generally strong because public benchmarks dominated.
Split integrity	57/8/0	65	57 studies were complete and 8 partial; partial scores reflected incomplete route/sequence leakage reporting.
Baseline adequacy	62/3/0	65	62 studies used adequate baselines and 3 used limited comparator sets.
Metric validity	65/0/0	65	All 65 studies reported a task-valid metric and evaluation protocol.
Corruption severity	9/14/0; N/A = 42	23	Among 23 applicable studies, 9 fully parameterized severity and 14 used partial or categorical levels; 42 were N/A.
Runtime evidence	15/43/7	65	15 studies were complete, 43 partial, and 7 unreported; end-to-end resource evidence remained uncommon.
Uncertainty/variability	6/30/29	65	Only 6 studies were complete; 30 were partial and 29 unreported, making this the weakest reporting domain.
Reproducibility	51/14/0	65	51 studies were complete and 14 partial; public data/codes were common, but some hardware-specific implementations were incomplete.

**Table 7:** Functional comparison of autonomous-vehicle sensing modalities. Synthesized from sensor surveys, benchmark datasets, and adverse-condition studies [6,7,8,9,10,11,12,13,14,15,16,17,18,19,20,21,22,23,24,25,26,27,28,29,30,31,32,36,37,44,82,83,84,85,86,87,88,89,90].

Modality	Principal Tasks	Strengths	Dominant Limitations
Camera/vision	Detection, segmentation, lanes, signs, depth	Dense semantics, low unit cost, mature datasets	Illumination, glare, blur, contamination, occlusion, depth ambiguity
LiDAR	3D detection, free space, mapping, localization	Metric range and geometry, strong 3D structure	Rain/fog/snow scattering, low reflectivity, blockage, cost, sparsity at range
Radar	Range-velocity estimation, detection, tracking, localization	Direct Doppler, long range, darkness/weather resilience	Angular sparsity, multipath, clutter, ghosts, interference, representation fragmentation
Ultrasonic	Parking and near-field obstacle sensing	Low cost and strong near-field utility	Short range, wide beam, cross-talk, temperature and surface-angle sensitivity
GNSS/IMU/odometry	Ego-state and localization	Global reference plus high-rate motion	Urban canyon, multipath, spoofing, bias drift, wheel slip
Thermal/event	Night, low light, high dynamic range, fast motion	Alternative spectral/temporal evidence	Limited semantic texture, specialized hardware, sparse datasets
V2X/cooperative sensing	Occlusion mitigation, extended horizon	Information beyond line of sight	Latency, trust, localization error, communication loss, security

**Table 8:** Comparison of camera families for automated driving. The table synthesizes original benchmark and method publications [9,10,11,12,13,14,20,27,29,30,31,32,70,71,72,73,74,75,76].

Camera Type	Distinctive Information	Primary Strengths	Safety-Relevant Limitations	Representative Sources
Monocular RGB	Dense color, texture, semantics	Low cost; scalable 360° coverage; signs, lights, lanes, classification	Scale ambiguity; illumination, glare, blur, contamination, domain shift	BDD100K, Cityscapes, camera-only BEV [14,27,70,71,72,73,74,75,76]
Stereo RGB	Disparity-based geometry plus semantics	Metric depth at short and medium range; passive sensing	Depth uncertainty grows with distance; texture and rectification dependence	KITTI and stereo-driving benchmarks [9,30]
Fisheye/surround view	Wide near-field coverage	Parking, curb, close-object and cross-traffic context	Strong distortion; reduced distant pixels; calibration sensitivity	Multi-camera benchmark systems [10,11,20]
Thermal infrared	Long-wave thermal contrast	Pedestrian cues in darkness; complementary to visible light	Temperature/emissivity dependence; limited color/text semantics	KAIST multispectral benchmark [29]
Event camera	Asynchronous brightness changes	High dynamic range; low latency; rapid motion and optical flow	Sparse static scenes; representation-window dependence; limited conventional texture	DSEC, EV-FlowNet, event-vision survey [30,31,32]

**Table 9:** LiDAR technology classes and safety-relevant evidence requirements. The comparison extends the physical and algorithmic evidence represented in LiDAR datasets, detectors, and adverse-weather studies [9,10,11,18,19,20,26,36,37,50,51,52,53,54,55,56,57].

LiDAR Architecture	Acquisition Characteristic	Potential Advantage	Principal Limitations and Failure Mechanisms	Evidence Considerations
Rotating mechanical	Sequential 360° or wide-FOV scanning	Mature geometry; broad coverage; established datasets	Moving parts; scan distortion; blockage; weather backscatter; mutual interference	Report rotation rate, channels, return mode, motion compensation, and cleaning.
MEMS scanning	Mirror-steered nonuniform or region-of-interest scan	Compact form; programmable scan patterns	Limited FOV; mirror dynamics; vibration and temperature sensitivity	Report scan pattern and whether benchmark density matches deployment.
Optical phased array	Electronic beam steering	No macroscopic moving parts; rapid steering	Side lobes, limited aperture/FOV, thermal and fabrication constraints	Hardware-specific claims require direct sensor characterization.
Flash LiDAR	Simultaneous full-frame illumination	No rolling scan distortion; useful near field	Range and power limits; ambient-light sensitivity; lower spatial resolution	Evaluate near-field detection and sunlight performance separately.
FMCW LiDAR	Coherent frequency-modulated ranging	Potential direct velocity and interference rejection	Complex coherent hardware; emerging datasets and validation	Avoid assuming equivalence with pulsed time-of-flight LiDAR.

**Table 10:** Automotive-radar data representations and their implications for fusion and safety assessment [10,16,17,21,22,23,24,25,65,66,67,68,79,80,81].

Radar Representation	Information Retained	Typical Tasks	Advantages	Limitations and Safety Implications	Representative Sources
Target list	Range, angle, Doppler, amplitude after proprietary detection	Tracking and adaptive cruise control	Low bandwidth; production-ready	Irreversible loss of weak/ambiguous returns; vendor-specific filtering	Automotive radar datasets [21]
Radar point cloud	Sparse detections with Doppler and reflectivity	3D detection, fusion, tracking	Convenient geometric interface	Sparse angular evidence; ghost targets; not equivalent to LiDAR	nuScenes/radar fusion [10,65,79]
Range-Doppler map	Range and radial-velocity energy	Motion detection, classification	Retains velocity structure	No direct azimuth without additional processing	Raw/tensor radar research [23,24,25]
Range-azimuth map	Spatial energy in polar coordinates	Detection and segmentation	Preserves angular structure	Velocity may be collapsed; clutter and sidelobes	CARRADA and radar detection [22,66]
4D radar cube/tensor	Range, azimuth, elevation, Doppler	3D detection, tracking, occupancy	Maximum retained information and imaging potential	High bandwidth, hardware specificity, calibration and interference sensitivity	3D radar cube and high-definition radar [24,25]
Temporal radar sequence	Consecutive frames or scans	Odometry, tracking, static-scene mapping	Exploits stable Doppler and long-range returns	Dynamic-object filtering and multipath remain critical	Radar odometry [80,81]

**Table 11:** Localization and cooperative sensing sources, failure mechanisms, and integrity indicators. The synthesis draws on representative visual-, LiDAR-, radar-, and inertial-estimation methods [80,81,82,83,84,85,86,87].

Source or Subsystem	Primary Contribution	Dominant Failure Mechanisms	Health/Integrity Indicators	Representative Mitigation and Sources
GNSS/RTK	Global position and time reference	Urban canyon, multipath, blockage, jamming, spoofing, correction loss	Satellite geometry, carrier residuals, innovation, correction age, protection level	Tightly couple with IMU, map, vision, LiDAR, or radar; reject inconsistent fixes.
IMU	High-rate angular velocity and specific force	Bias, scale-factor error, saturation, temperature, vibration, integration drift	Bias estimate, temperature, saturation flag, innovation and covariance growth	Online calibration, redundant IMUs, motion constraints, periodic absolute correction [83,86,87].
Wheel odometry	Vehicle speed and kinematic constraints	Slip, tire-radius change, wheel lift, encoder fault	Wheel-speed consistency, yaw-rate residual, slip estimate	Fuse with IMU and exteroceptive odometry; detect low-friction conditions.
Visual-inertial odometry	Metric local motion from image and IMU	Texture loss, blur, illumination, dynamic scenes, calibration error	Feature count, reprojection error, inertial residual, estimator covariance	OKVIS, VINS-Mono, OpenVINS, ORB-SLAM3 [83,84,85,86,87].
LiDAR-inertial odometry	Geometric local motion and mapping	Sparse geometry, weather, blockage, scan distortion	Registration fitness, map residual, point occupancy, IMU consistency	LIO-SAM and multi-hypothesis mapping [82].
Radar odometry	Velocity-rich motion in darkness/adverse weather	Multipath, dynamic objects, low angular resolution	Static-return ratio, Doppler residual, registration consistency	Learned keypoints and probabilistic radar odometry [80,81].
Cooperative/V2X perception	Beyond-line-of-sight objects and infrastructure context	Latency, packet loss, sender localization error, authentication and trust	Message age, sender covariance, consistency with local tracks, signature status	Preserve provenance; gate remote evidence; never overwrite strong local evidence without consistency checks.

**Table 12:** Fusion levels, representations, and safety implications. Synthesized from multisensor-fusion theory and representative camera–LiDAR–radar architectures [8,44,58,59,60,61,62,63,64,65,66,67,68,69,70,71,72,73,74,75,76,77,78,79].

Fusion Level	Definition	Advantages	Safety-Relevant Limitations
Early/data-level	Raw or lightly processed measurements combined before feature extraction.	Maximum information retention; joint denoising possible.	High bandwidth; calibration-sensitive; modality-specific sampling mismatch.
Feature/middle-level	Learned features fused in image, point, voxel, range-view, or BEV space.	Strong task performance; flexible attention and weighting.	Can hide sensor validity; representation may privilege one modality.
Late/decision-level	Independent detections, tracks, occupancy, or hypotheses combined.	Modular; easier provenance and fault isolation.	Cannot recover information discarded by unimodal branches.
Track/state-level	Probabilistic object states or ego-state estimates fused over time.	Explicit uncertainty and temporal consistency.	Dependent on association and model assumptions.
Reliability-aware fusion	Feature or decision weights conditioned on sensor health and task observability.	Supports missing modalities and graceful degradation.	Requires trustworthy health labels and calibrated confidence.

**Table 13:** Representative multimodal fusion architectures and reliability questions that should accompany nominal benchmark accuracy [58,59,60,61,62,63,64,65,66,67,68,69,77,78,79].

Representative Architecture	Modalities and Representation	Fusion Mechanism	Strength Relevant to Sensing	Reliability Limitation Requiring Explicit Testing
MV3D [58]	Camera + LiDAR; multi-view proposals	Proposal and feature fusion across BEV/front/image views	Early demonstration of complementary 3D views	Projection and proposal dependence; limited missing-modality evidence.
AVOD [59]	Camera + LiDAR; BEV/image features	Feature aggregation for proposals and boxes	Efficient joint proposals	Calibration and image-quality dependence.
Deep Continuous Fusion [60]	Camera + LiDAR	Continuous geometric feature fusion	Dense image-point association	Association errors under miscalibration or timing drift.
PointPainting [61]	Camera semantics painted onto LiDAR points	Sequential semantic augmentation	Simple use of image segmentation in 3D detection	Camera errors are transferred into the point cloud.
TransFusion [62]	Camera + LiDAR; object queries	Transformer-based soft association	Flexible cross-modal association	Attention weights are not automatically calibrated reliability.
BEVFusion variants [63,64]	Camera + LiDAR; common BEV	Modality-specific encoders and BEV fusion	Planning-aligned representation; strong benchmark performance	Depth projection, compute cost, and corrupted-modality behavior.
CenterFusion [65]	Camera + radar	Radar association with center-based image detection	Adds direct velocity and range cues	Camera detector remains a structural dependency.
RODNet/radar-camera methods [66,67,68]	Radar tensors/points + images	Cross-modal supervision or feature fusion	Improves radar semantics and distant-object detection	Representation- and calibration-specific transfer.
Multi-modal Fusion Transformer [69]	Camera + LiDAR; end-to-end driving	Transformer fusion for perception/control	Joint temporal and task context	End-to-end behavior can obscure sensor provenance and failure cause.
RadarNet/DeepFusion/FusionPainting [77,78,79]	LiDAR/camera/radar combinations	Adaptive attention or deep feature fusion	Rich multisensor complementarity	Needs physically realistic missing/corrupted-modality evaluation.

**Table 14:** Failure modes, diagnostic evidence, and example operational responses. Synthesized from safety standards, adverse-weather experiments, robustness benchmarks, and fault-injection studies [18,19,28,33,34,35,36,37,38,39,40,41,42,43].

Modality	Representative Degradation	Health Evidence	Possible Operational Response
Camera	Blur, glare, low light, over/under-exposure, lens droplets, mud, blockage, rolling-shutter/timing error	Quality metrics; obstruction segmentation; temporal consistency; cross-modal residuals	De-weight image features; restrict speed/ODD; rely on range sensors; clean lens; fallback
LiDAR	Fog/rain/snow scattering, low reflectivity, aperture blockage, missing rings, timing drift, interference	Return rate; ring continuity; intensity distribution; scan overlap; sector occupancy	Filter clutter; de-weight sectors; increase radar dependence; restrict range/speed; clean/heating
Radar	Interference, clutter, multipath, ghost targets, misalignment, sensitivity loss	Noise floor; spectral occupancy; target statistics; ego-motion residuals; map consistency	Adaptive filtering; lower track confidence; require cross-modal confirmation; ODD restriction
Ultrasonic	Cross-talk, temperature drift, wide-beam ambiguity, blocked transducer	Echo strength; diagnostic pulse; consistency across adjacent sensors	Disable automated parking function; reduce maneuver speed; request driver takeover
GNSS	Multipath, blockage, jamming, spoofing, correction loss	RAIM/integrity; innovation tests; signal metrics; map and inertial consistency	Switch to local localization; expand uncertainty; restrict route/ODD; minimum-risk stop
IMU/odometry	Bias drift, saturation, vibration, scale error, wheel slip	Bias/temperature model; residuals; multisensor innovation; wheel-speed consistency	Reinitialize; increase covariance; use vision/LiDAR/radar odometry; restrict dynamics
V2X	Latency, packet loss, false sender state, localization error	Timestamp/provenance; authentication; plausibility and local-sensor consistency	Treat as advisory; age-out messages; reduce cooperative dependence

**Table 15:** Examples of sensor-health indicators, diagnostic hypotheses, cross-checks, and operational responses. The table synthesizes safety standards and fault/robustness studies [18,19,28,33,34,35,36,37,38,39,40,41,42,43]. The merged table also provides traceability from the included evidence to the proposed reliability-aware architecture. The final column distinguishes demonstrated results from indirect support and proposed design principles.

**Sensor/Subsystem**	**Observable Health Indicators**	**Degradation Hypotheses**		**Cross-Checks**		**Potential Operational Response**
RGB/stereo camera	Exposure histogram, saturation, blur, noise, obstruction mask, frame age, stereo residual	Glare, darkness, blur, droplets, mud/snow, dropped/stale frames, misalignment		Overlapping cameras, LiDAR/radar object residuals, lane/map consistency		Reduce speed; disable lane-change automation; clean lens; rely on radar/LiDAR if residual observability is sufficient.
LiDAR	Point count by ring/sector, intensity distribution, near-return clutter, scan overlap, packet age	Aperture blockage, fog/rain/snow attenuation, ring failure, interference, timing error		Radar/camera tracks, map occupancy, ego-motion registration residual		Restrict speed/range; activate heater/washer; exclude affected sector; minimum-risk maneuver if stopping horizon is unsupported.
Radar	Noise floor, received power, spectral occupancy, target count, range-Doppler statistics, temperature	Interference, sensitivity loss, misalignment, ghost targets, packet loss		Camera/LiDAR association, ego-motion and map consistency		Reweight radar; suppress affected bins/sectors; increase headway; restrict high-speed operation.
GNSS/RTK	Satellite geometry, correction age, pseudorange/carrier residual, protection level	Blockage, multipath, correction loss, jamming, spoofing		IMU, wheel, map, visual/LiDAR/radar odometry		Reject fix; increase localization uncertainty; constrain route or request minimum-risk stop.
IMU/wheel odometry	Bias and scale estimates, saturation, temperature, wheel-speed consistency, slip estimate	Bias ramp, vibration, saturation, encoder fault, tire slip		Exteroceptive odometry and vehicle-dynamics model		Reduce reliance; enlarge covariance; limit aggressive maneuvers.
Fusion/world model	Cross-modal residuals, track provenance, observation age, uncertainty calibration, missing sectors	Miscalibration, desynchronization, corrupted high-confidence modality, stale tracks		Independent sensor-health layer and scenario requirements		Dynamic gating; preserve uncertainty; ODD restriction; transition or minimum-risk maneuver [33,34,35,40,41,42,43].
**Architecture Function**	**Direct Reviewed Evidence**		**Indirect/Normative Support**		**Evidence Status and Claim Boundary**	
Sensor-health estimation	Confidence monitoring and fault-injection evaluation [40,41,42].		Physical corruption and robustness studies [36,37,38,39].		Direct component-level support; limited sensor diversity and field validation.	
Health-conditioned modality reweighting	No included study validates the complete closed-loop function.		Fusion/association/robustness methods [62,63,64,65,66,67,68,69,77,78,79].		Proposed synthesis principle; fusion gains do not prove diagnostic validity.	
Uncertainty and provenance in the world model	Partial evidence from probabilistic estimation and confidence studies [7,40,81,87].		Safety-case and fusion principles [33,34,35,43,44].		Partially supported; end-to-end provenance is rarely evaluated.	
ODD restriction	Sparse simulation/system evidence [41,42].		SAE J3016, ISO 26262/21448, and UL 4600 [1,33,34,35].		Normative/synthesis-level recommendation, not a demonstrated corpus-wide outcome.	
Transition/minimum-risk behavior	Fault-to-behavior simulation evidence is limited [42].		Safety and fallback reasoning [1,33,34,35,43].		Proposed operational response; field effectiveness remains unvalidated.	

**Table 16:** Representative datasets and benchmark limitations. Dataset properties and limitations were synthesized from the original dataset publications [9,10,11,12,13,14,15,16,17,18,19,20,21,22,23,24,25,26,27,28,29,30].

Dataset	Sensor Suite	Primary Use	Critical Limitation
KITTI [9]	Stereo RGB, LiDAR, GNSS/IMU	Detection, tracking, odometry	Foundational benchmark; limited geography and weather diversity.
nuScenes [10]	6 cameras, 5 radars, LiDAR, GNSS/IMU	3D detection, tracking, prediction	Multimodal and synchronized; relatively sparse LiDAR and urban focus.
Waymo Open Dataset [11]	Multi-camera, multiple LiDARs	3D detection, tracking, motion	Large scale and rich labels; limited raw radar access.
Argoverse 1/2 [12,13]	Cameras, LiDAR, maps	Tracking, forecasting, 3D detection	Detailed maps and urban diversity; sensor suites differ by version.
BDD100K [14]	Front camera, GPS/IMU metadata	Detection, lanes, drivable area, tracking	Large visual diversity; limited direct range sensing.
Oxford RobotCar [15]	Cameras, LiDAR, radar, GPS/INS	Localization and long-term autonomy	Repeated routes and seasons; legacy hardware and UK geography.
Oxford Radar RobotCar [16]	Navtech radar plus localization sensors	Radar odometry/localization	Raw radar and long-term routes; limited object annotations.
RADIATE [17]	Radar, LiDAR, cameras, GNSS	Detection in adverse weather	Weather diversity; smaller scale than major camera/LiDAR datasets.
Seeing Through Fog [18]	Camera, gated camera, thermal, LiDAR, radar	Detection and weather analysis	Controlled and real fog; specialized sensor suite.
CADC [19]	Cameras, LiDAR, GNSS/INS	3D detection in winter	Snow and cold conditions; geographic concentration.
PandaSet [20]	Cameras, mechanical and solid-state LiDAR	Detection and segmentation	Diverse LiDAR configurations; moderate corpus size.
View of Delft [21]	Camera, 3+1D radar, LiDAR	Radar-camera 3D detection	High-resolution radar; limited city and sensor configuration.
CARRADA/RADIal [22,25]	Raw radar tensors and cameras	Detection, segmentation, free space	Supports raw radar learning; smaller scenarios and taxonomies.
SemanticKITTI [26]	LiDAR sequences	Semantic scene completion/segmentation	Strong point labels; inherited KITTI environment limits.
ACDC [28]	RGB images in fog, night, rain, snow	Semantic segmentation	Real adverse conditions; camera-only.
KAIST Multispectral [29]	Aligned RGB and thermal	Pedestrian detection	Day/night thermal evidence; limited task diversity.
DSEC [30]	Stereo event cameras, frames, LiDAR, GPS	Stereo, flow, segmentation	High-rate event data; specialized processing requirements.
CARLA/AirSim [47,48]	Configurable simulated sensors	Scenario generation and fault injection	Controllable and scalable; simulation-to-real gap.
LiDARsim [49]	Data-driven LiDAR simulation	Detection training and simulation	Improved realism; depends on source data and model validity.

**Table 17:** Recommended evaluation metrics for reliability-centred autonomous-vehicle sensing. The table synthesizes benchmark practice, robustness evidence, and safety-case requirements [9,10,11,12,13,14,15,16,17,18,19,20,21,22,23,24,25,26,27,28,29,30,33,34,35,36,37,38,39,40,41,42,43].

Evaluation Objective	Recommended Metrics	Required Stratification or Protocol	Safety Interpretation
2D/3D detection	Precision, recall, AP/mAP, false negatives per distance, localization error	Class, distance, object size, occlusion, weather, sensor health	Report missed-object risk near stopping and collision horizons, not aggregate AP alone.
Segmentation/occupancy	IoU, class IoU, calibration, occupied/free-space false rate	Near/far range, road boundary, adverse condition, unknown space	Unknown or unobserved space should not be forced into free space.
Tracking	MOTA/HOTA, ID switches, fragmentation, track latency, false-track lifetime	Dynamic/static objects, occlusion, sensor loss and recovery	Long-lived false tracks and delayed re-acquisition can be more hazardous than frame-level errors.
Localization/odometry	RMSE, drift, percentile/tail error, integrity risk, protection level, time to alert	Urban canyon, tunnel, weather, dynamic scene, spoofing/jamming	Average error must be supplemented by hazardous misleading-information probability.
Uncertainty	Expected calibration error, NLL, Brier score, coverage versus interval width	Clean and each corruption severity; in-domain and cross-domain	Confidence should remain calibrated when sensors degrade.
Sensor-health monitoring	Detection rate, false-alarm rate, time to detection, severity at detection, recovery time	Abrupt/gradual faults, partial/full blockage, common-cause failures	Health detection must occur before residual observability becomes unsafe.
Real-time system	Sensor-to-plan latency, jitter, deadline misses, throughput, power, temperature	Complete pipeline and worst-case load; prolonged operation	Inference time alone does not establish timely perception.
Graceful degradation	Performance-severity curve, residual ODD, safe-speed envelope, transition/minimum-risk success	Fault combinations and scenario-specific requirements	Quantifies whether the vehicle remains within a defensible operating envelope.

**Table 18:** Deployment and safety-case evidence expected for reliability-centered sensor architectures [6,7,33,34,35,40,41,42,43].

Assurance Dimension	Evidence Expected from a Deployment-Oriented Study	Common Reporting Weakness
Coverage and ODD	Sensor placement, overlap, blind zones, required detection horizon, weather/lighting limits	Generic “360° coverage” without range, occlusion, or task requirements.
Independence and common cause	Failure-dependency analysis across power, compute, clocks, cleaning, mounting, and environment	Counting multiple sensors as redundant despite shared failure mechanisms.
Calibration and synchronization	Acceptance tolerances, online monitoring, time-to-detection, recalibration process	Only initial calibration values or dataset-provided transforms.
Latency and compute	Sensor exposure/scan, transfer, preprocessing, fusion, tracking, publication, worst-case jitter	Reporting neural-network inference time only.
Power and thermal behavior	Steady-state and peak power, cleaning/heating demand, thermal throttling, low-energy mode	No prolonged-operation or derating evidence.
Uncertainty and health	Calibration under corruption, false alarms, detection latency, provenance, residual observability	Confidence scores reported without calibration or operational thresholds.
Fallback and recovery	Safe-speed envelope, ODD restriction, transition logic, minimum-risk maneuver, cleaning/reset recovery	Fault detection evaluated without vehicle-level response.
Maintainability	Sensor replacement tolerance, calibration after service, contamination management, diagnostic logs	Prototype performance without lifecycle and service considerations.
Reproducibility	Hardware IDs, firmware, preprocessing, splits, seeds, code/data, failure-injection parameters	Insufficient detail to repeat or compare the result.

**Table 19:** Research agenda for reliability-centered autonomous-vehicle sensing. Priorities were derived from cross-modal evidence gaps and safety-assurance requirements [6,7,18,19,28,33,34,35,36,37,38,39,40,41,42,43].

Priority	Required Research Action	Minimum Evidence Standard
Physically grounded corruptions	Create severity-controlled datasets for lens contamination, partial LiDAR blockage, radar interference, timestamp drift, GNSS spoofing, and calibration movement.	Corruption model linked to measurable physical parameters and validated against road data.
Task-level observability	Translate sensor quality into observable range, object class, region, and maneuver capability.	Calibration curves from health indicator to task failure probability.
Uncertainty and provenance	Preserve which sensor, time, and transformation support each world-model hypothesis.	Calibrated probabilistic outputs and auditable data lineage.
Graceful degradation	Evaluate speed reduction, headway increase, route restriction, transition request, and minimal-risk behavior after fault onset.	System-level safety metrics rather than perception accuracy alone.
Cross-dataset and cross-hardware transfer	Test models across cities, weather, sensor generations, and calibration configurations.	Predefined external validation with no target-domain tuning.
Common-cause failure analysis	Model shared power, network, clock, mount, environmental, and software dependencies.	Fault trees or Bayesian networks connected to empirical sensor evidence.
Real-time and energy reporting	Measure end-to-end latency, bandwidth, memory, power, thermal behavior, and worst-case timing.	Embedded-platform measurements under sustained load.
Open health labels	Annotate sensor state, visibility, blockage, interference, and confidence validity.	Benchmark tasks for diagnosis, not only perception.
Security-safety co-engineering	Evaluate spoofing, injection, jamming, and authenticated-but-wrong cooperative data.	Joint threat, fault, and fallback scenarios.
Reproducible systematic evidence	Publish database exports, screening decisions, extraction forms, and query histories.	PRISMA-S-compliant supplement and living-review updates.

**Table 20:** Minimum reporting checklist for autonomous-vehicle sensor, fusion, degradation, and health-monitoring studies. The checklist consolidates the methodological and assurance gaps identified in the review [6,7,33,34,35,40,41,42,43,45,46].

Checklist Item	Minimum Information to Report
Sensor specification	Model, wavelength/frequency, resolution, frame/scan rate, range, FOV, placement, overlap, firmware, preprocessing.
Timing and calibration	Clock source, timestamps, scan/exposure duration, latency, motion compensation, intrinsics/extrinsics, tolerances, monitoring.
Dataset and split	Source, geography, weather, route, sample/sequence count, train/validation/test separation, leakage controls.
Degradation model	Physical mechanism, affected sector/channel, severity scale, abrupt or gradual profile, validation against real data.
Baselines and ablations	Each unimodal baseline, health-unaware fusion, sensor removal, partial corruption, common-cause combinations.
Metrics	Task accuracy, uncertainty calibration, health-detection probability, false alarms, time to detection, latency, power, recovery.
Operational consequence	Residual ODD, speed/headway restriction, disabled maneuvers, transition or minimum-risk maneuver.
Reproducibility	Code, models, data or access route, configurations, seeds, hardware/software versions, evaluation scripts.

## Data Availability

The final executed search strings and database counts are reported in Table 3; complete PRISMA accounting and exclusion reasons are reported in Figure 2 and Section 3.1; and the included-study evidence matrix is provided in Appendix A. The complete study-level methodological quality appraisal is provided as Supplementary File S1, and the completed PRISMA 2020 and PRISMA-S reporting checklists are provided as Supplementary File S2. The row-level screening decision log and original database exports are available from the corresponding author on reasonable request; they are not publicly deposited because they contain licensed bibliographic metadata from subscription databases. The review was retrospectively registered in PROSPERO on 30 July 2026 (CRD420261465869); no separate OSF registration was used.

## Supplementary Materials

The following supporting information can be downloaded at https://www.mdpi.com/article/10.3390/s26165316/s1: Supplementary File S1: Study-level methodological quality appraisal of the 65 included primary studies [9,10,11,12,13,14,15,16,17,18,19,20,21,22,23,24,25,26,27,28,29,30,31,36,37,38,39,40,41,42,49,52,53,54,55,56,57,58,59,60,61,62,63,64,65,66,67,68,69,70,71,72,73,74,75,76,77,78,79,80,81,82,83,84,87]; Supplementary File S2: Completed PRISMA 2020 and PRISMA-S reporting checklists [45,46].

## Abbreviations

**Abbreviation**	**Definition**
ACDC	Adverse Conditions Dataset with Correspondences
ADAS	Advanced Driver Assistance System
AI	Artificial Intelligence
ANN	Artificial Neural Network
AV	Autonomous Vehicle
BEV	Bird’s-Eye View
CADC	Canadian Adverse Driving Conditions Dataset
CNN	Convolutional Neural Network
DNN	Deep Neural Network
DOD	Department of Defense
DSEC	Driving Stereo Event Camera Dataset
FMCW	Frequency-Modulated Continuous Wave
FOV	Field of View
GNSS	Global Navigation Satellite System
GPS	Global Positioning System
HD	High Definition
IMU	Inertial Measurement Unit
INS	Inertial Navigation System
IoU	Intersection over Union
ISO	International Organization for Standardization
KITTI	Karlsruhe Institute of Technology and Toyota Technological Institute Dataset
LiDAR	Light Detection and Ranging
mAP	Mean Average Precision
ML	Machine Learning
ODD	Operational Design Domain
OSF	Open Science Framework
PCA	Principal Component Analysis
PRISMA	Preferred Reporting Items for Systematic Reviews and Meta-Analyses
PRISMA-S	Preferred Reporting Items for Systematic Reviews and Meta-Analyses—Search Extension
RADAR	Radio Detection and Ranging
RGB	Red–Green–Blue
RIS	Research Information Systems (citation exchange format)
RTK	Real-Time Kinematic
RQ	Research Question
SAE	Society of Automotive Engineers
SLAM	Simultaneous Localization and Mapping
SOTIF	Safety of the Intended Functionality (ISO 21448)
TS	Topic Search (Web of Science)
UAV	Unmanned Aerial Vehicle
UL	Underwriters Laboratories
V2I	Vehicle-to-Infrastructure
V2V	Vehicle-to-Vehicle
V2X	Vehicle-to-Everything
WoS	Web of Science

## Appendix A. Final Evidence Matrix of Included Primary Studies

**Table A1:** Final evidence matrix (65 included primary studies). Each row corresponds to one study in the final PRISMA set and is linked to the reference list [9,10,11,12,13,14,15,16,17,18,19,20,21,22,23,24,25,26,27,28,29,30,31,36,37,38,39,40,41,42,49,52,53,54,55,56,57,58,59,60,61,62,63,64,65,66,67,68,69,70,71,72,73,74,75,76,77,78,79,80,81,82,83,84,87].

Study	Sensors	Task/Type	Main Contribution	Key Limitation for Safety Interpretation
Geiger et al. (2012) [9]	Camera + LiDAR + GNSS/IMU	Benchmark/dataset	KITTI calibration and annotated road scenes	Strong benchmark standardization; limited geography and weather.
Caesar et al. (2020) [10]	Camera + radar + LiDAR	Dataset	nuScenes 360° multimodal benchmark	Rich multimodal labels; relatively sparse LiDAR.
Sun et al. (2020) [11]	Camera + LiDAR	Dataset	Large-scale 3D detection and tracking corpus	Limited public raw radar.
Chang et al. (2019) [12]	Camera + LiDAR + maps	Tracking/forecasting dataset	Argoverse map-rich urban sequences	City and sensor-specific priors.
Wilson et al. (2021) [13]	Camera + LiDAR + maps	Dataset	Argoverse 2 diversified motion and perception tasks	Hardware/version differences complicate comparison.
Yu et al. (2020) [14]	Camera	Dataset	Large-scale diverse driving video labels	No direct metric-range sensor.
Maddern et al. (2017) [15]	Camera + LiDAR + radar + GNSS/INS	Long-term localization dataset	Repeated route across seasons and weather	Legacy configuration and single metropolitan region.
Barnes et al. (2020) [16]	Radar + localization sensors	Radar dataset	Raw radar odometry and localization	Object-level labels limited.
Sheeny et al. (2021) [17]	Radar + LiDAR + camera	Adverse-weather dataset	Radar-focused detection in weather	Smaller scale than mainstream datasets.
Bijelic et al. (2020) [18]	Camera + gated + thermal + LiDAR + radar	Adverse weather	Controlled/real fog multimodal evaluation	Specialized sensor suite.
Pitropov et al. (2021) [19]	Camera + LiDAR + GNSS/INS	Winter dataset	Snow and cold-weather 3D detection	Regional concentration.
Xiao et al. (2021) [20]	Camera + dual LiDAR types	Dataset	PandaSet multimodal sensor suite	Moderate corpus size.
Meyer and Kuschk (2019) [21]	Camera + radar + LiDAR	Radar–camera detection	View of Delft high-resolution radar	Limited scenes and configuration.
Ouaknine et al. (2021) [22]	Camera + raw radar	Dataset	CARRADA range-angle-Doppler labels	Small scenario and class set.
Major et al. (2019) [23]	Automotive radar	3D detection	Learning from range–azimuth–Doppler tensors	Hardware-specific representation.
Palffy et al. (2020) [24]	3D radar	Road-user detection	CNN learning on radar cube	Limited public scale.
Rebut et al. (2022) [25]	Raw high-definition radar + camera	Multi-task perception	Joint detection and free-space learning	Specialized radar hardware.
Behley et al. (2019) [26]	LiDAR	Semantic segmentation dataset	SemanticKITTI sequence labels	Inherited KITTI domain limits.
Cordts et al. (2016) [27]	Camera	Semantic segmentation dataset	Fine urban pixel labels	Good weather and European-city bias.
Sakaridis et al. (2021) [28]	Camera	Adverse-condition segmentation	Real fog, night, rain, snow	No range sensing.
Hwang et al. (2015) [29]	RGB + thermal	Pedestrian detection	KAIST aligned multispectral benchmark	Specialized task and geography.
Gehrig et al. (2021) [30]	Event + frame + LiDAR	Dataset	DSEC stereo/event driving	Specialized processing and limited adoption.
Zhu et al. (2018) [31]	Event camera	Optical flow/ego-motion	Learning event-based flow	Limited semantics in static regions.
Hahner et al. (2022) [36]	LiDAR	Snowfall degradation	Severity-controlled LiDAR snowfall simulation	Synthetic particle model requires validation across hardware and real storms.
Hahner et al. (2021) [37]	LiDAR	Fog degradation	Physically parameterized fog simulation on real point clouds	Transfer to different wavelengths, scanners, and natural fog is conditional.
Manivasagam et al. (2020) [49]	LiDAR simulation	Simulation/training	Data-driven LiDARsim	Validity depends on source and rendering model.
Michaelis et al. (2019) [38]	Camera	Corruption robustness	Object-detection robustness benchmark for driving scenes	Generic corruptions are not equivalent to physical sensor impairment.
Hendrycks and Dietterich (2019) [39]	Camera/general vision	Corruption benchmark	Standardized common-corruption severity framework	Not vehicle-specific and does not model calibrated sensor-health states.
Zhou and Tuzel (2018) [52]	LiDAR	3D detection	VoxelNet end-to-end voxel features	Computationally demanding.
Yan et al. (2018) [53]	LiDAR	3D detection	SECOND sparse convolution detector	Voxelization and sparsity assumptions.
Lang et al. (2019) [54]	LiDAR	3D detection	PointPillars efficient pillar encoding	Vertical detail compressed.
Shi et al. (2019) [55]	LiDAR	3D detection	PointRCNN point-based proposals	Computational complexity.
Shi et al. (2020) [56]	LiDAR	3D detection	PV-RCNN point-voxel fusion	High memory and compute.
Yin et al. (2021) [57]	LiDAR	3D detection/tracking	CenterPoint center-based BEV detection	Still vulnerable to sparse/blocked input.
Chen et al. (2017) [58]	Camera + LiDAR	3D detection	MV3D multi-view fusion	Calibration-sensitive and complex.
Ku et al. (2018) [59]	Camera + LiDAR	3D detection	AVOD feature aggregation	Fixed projection assumptions.
Liang et al. (2018) [60]	Camera + LiDAR	3D detection	Continuous fusion across resolutions	Requires precise correspondence.
Vora et al. (2020) [61]	Camera + LiDAR	3D detection	PointPainting semantic augmentation	Camera errors propagate into points.
Bai et al. (2022) [62]	Camera + LiDAR	3D detection	TransFusion attention-based association	Health uncertainty not explicit.
Liu et al. (2023) [63]	Camera + LiDAR	Multi-task BEV fusion	Unified multi-task BEV representation	Compute and calibration dependence.
Liang et al. (2022) [64]	Camera + LiDAR	Robust LiDAR-camera BEV fusion	Robust BEV fusion under simulated LiDAR malfunction	Fault model is simulated and benchmark-specific.
Nabati and Qi (2021) [65]	Camera + radar	3D detection	CenterFusion radar velocity/depth cues	Radar sparsity and association errors.
Kim et al. (2021) [66]	Radar	Object detection	RODNet temporal radar learning	Dataset/hardware dependence.
Chadwick et al. (2019) [67]	Camera + radar	Distant vehicle detection	Radar-guided visual proposals	Limited task/classes.
Meyer and Kuschk (2019) [68]	Camera + radar	3D detection	Radar–camera deep fusion	Limited radar resolution.
Prakash et al. (2021) [69]	Camera + LiDAR	End-to-end driving	TransFuser transformer-based sensor fusion	CARLA-based evidence and opaque end-to-end failure attribution.
Li et al. (2022) [70]	Multi-camera	BEV perception	BEVFormer spatiotemporal transformer	Depth and calibration uncertainty.
Philion and Fidler (2020) [71]	Multi-camera	BEV perception	Lift–Splat–Shoot view transformation	Depth distribution errors.
Wang et al. (2022) [72]	Multi-camera	3D detection	DETR3D 3D queries	Large compute and camera dependence.
Liu et al. (2022) [73]	Multi-camera	3D detection	PETR positional embedding	Calibration sensitivity.
Liu et al. (2023) [74]	Multi-camera	3D detection	PETRv2 temporal extensions	Temporal drift and compute.
Huang et al. (2021) [75]	Multi-camera	BEV detection	BEVDet efficient view transformation	Nominal image quality assumptions.
Li et al. (2023) [76]	Multi-camera	BEV depth/detection	BEVDepth explicit depth supervision	Depth labels and domain transfer.
Xu et al. (2021) [77]	Camera + LiDAR	3D detection	FusionPainting adaptive semantic fusion	Camera semantics can mislead.
Li et al. (2022) [78]	Camera + LiDAR	3D detection	DeepFusion alignment-aware fusion	Robustness evidence limited.
Yang et al. (2020) [79]	Radar + LiDAR	3D detection	RadarNet long-range radar fusion	Association and hardware dependence.
Barnes et al. (2020) [80]	Radar	Odometry/localization	Learned robust radar keypoints	Dynamic clutter and route dependence.
Burnett et al. (2021) [81]	Radar	Odometry	Probabilistic radar odometry	Multipath and generalization.
Shan et al. (2020) [82]	LiDAR + IMU	Localization/SLAM	LIO-SAM factor-graph smoothing	LiDAR structure and calibration required.
Qin et al. (2018) [83]	Camera + IMU	Visual–inertial odometry	VINS-Mono tightly coupled estimator	Visual degradation and initialization.
Campos et al. (2021) [84]	Camera + IMU	SLAM	ORB-SLAM3 multi-map visual-inertial SLAM	Texture and illumination dependence.
Geneva et al. (2020) [87]	Camera + IMU	State estimation	OpenVINS reproducible estimator	Camera/IMU calibration critical.
Jeong et al. (2023) [40]	LiDAR + chassis sensors	Fault detection	Confidence-level evaluation for AV perception	Limited scenarios and sensor diversity.
Tian et al. (2025) [41]	Camera + LiDAR fusion	Fault injection/testing	FADE system-level fault tolerance testing	Preprint and platform-specific.
Matos et al. (2025) [42]	Simulated sensor suite	Fault injection	Vehicle-behavior impact of sensor failures	Simulation assumptions require physical validation.
