# Supplementary material for: From Modality Performance to Graceful Degradation: A PRISMA 2020 Systematic Review of Sensor Architectures for Autonomous Vehicles
Source: Sensors (Basel). 2026 Aug 21;26(16):5316. doi: 10.3390/s26165316 (PMC13517109; doi:10.3390/s26165316)
Supplement: Supplementary file 1 [file sensors-26-05316-s001.zip › sensors-4503111-supplementary-File S2.pdf]

PRISMA element	Implementation	Location
Title	Identifies the report as a systematic review.	Title page
Abstract	Structured summary of objective, sources, methods, results, limitations, and conclusions.	Abstract
Rationale	Explains fragmentation of modality and reliability evidence.	Section 1
Objectives	Defines RQ1–RQ5.	Section 1
Eligibility criteria	Inclusion/exclusion rules.	Section 2.2; Table 1
Information sources	Databases and citation searching.	Section 2.3
Search strategy	Modular reproducible query blocks and database-specific syntax.	Section 2.3; Tables 2 and 3
Selection process	Two-reviewer screening, disagreement resolution, and auditable deduplication.	Sections 2.4 and 2.5; Table 4
Data collection	Structured extraction fields with independent verification.	Section 2.6
Data items	Sensors, tasks, failures, metrics, runtime, uncertainty, and operational response.	Section 2.6
Risk of bias/quality	Ten-item engineering appraisal with sensitivity interpretation.	Section 2.7; Table 5
Synthesis methods	Structured narrative synthesis, heterogeneity framework, and certainty interpretation.	Section 2.8
Study selection	PRISMA flow with transparent pilot counts pending replacement by verified database-derived counts.	Section 2.5; Figure 2
Study characteristics	Evidence matrix and dataset table.	Sections 3 and 7; Appendix A
Results of individual studies	Modality and architecture synthesis.	Sections 3–7
Results of syntheses	Cross-modal conclusions and research gaps.	Sections 8–9
Reporting bias	Addressed through neutral search terms and negative-result sensitivity.	Sections 2.3 and 10
Certainty of evidence	Interpreted through the quality checklist, heterogeneity, replication, and safety relevance.	Sections 2.7, 2.8, and 10
Discussion	General interpretation, limitations, and implications.	Sections 8–10
Registration/protocol	OSF registration to be added before submission.	Section 2.1
Support	Funding statement.	Back matter
Competing interests	Conflict statement.	Back matter

PRISMA element	Implementation	Location
Availability	Search exports and extraction matrix to be deposited.	Data Availability Statement
